# Not All Phrases Are Equally Attractive: Experimental Evidence for Selective Agreement Attraction Effects

**DOI:** 10.3389/fpsyg.2018.01566

**Published:** 2018-08-28

**Authors:** Dan Parker, Adam An

**Affiliations:** Linguistics Program, Department of English, College of William & Mary, Williamsburg, VA, United States

**Keywords:** sentence comprehension, encoding, retrieval, agreement attraction, self-paced reading

## Abstract

Research on memory retrieval during sentence comprehension suggests that similarity-based interference is mediated by the grammatical function of the distractor. For instance, Van Dyke and McElree (2011) observed interference during retrieval for subject-verb thematic binding when the distractor occurred as an oblique argument inside a prepositional phrase (PP), but not when it occurred as a core argument in direct object position. This contrast motivated the proposal that constituent encodings vary in the distinctiveness of their memory representations based on an argument hierarchy, which makes them differentially susceptible to interference. However, this hypothesis has not been explicitly tested. The present study uses an interference paradigm involving agreement attraction (e.g., Wagers et al., 2009) to test whether the argument status of the distractor determines susceptibility to interference. Results from two self-paced reading experiments show a clear contrast: agreement attraction is observed for oblique arguments (e.g., PP distractors), but attraction is nullified for core arguments (i.e., direct object and subject distractors). A follow-up experiment showed that this contrast cannot be reduced to the syntactic position of the distractor, favoring an account based on the semantic properties of the distractor. These findings support the proposal that interference is mediated by the argument status of the distractor and extend previous results by showing that the effect generalizes to a broader set of syntactic contexts and a wider range of syntactic dependencies. More generally, these results motivate a more nuanced account of real-time agreement processing that depends on both retrieval and encoding mechanisms.

## Introduction

Sentence comprehension routinely relies on memory retrieval mechanisms to establish grammatical dependencies among the words and phrases in a sentence. For instance, to relate the verb *were* in (1) to its subject to establish subject-verb number agreement, memory retrieval mechanisms must access the encoding of the plural subject *girls* and ignore featurally similar information in non-target positions, such as the embedded plural noun *boys*.

(1) **The girls_**PL**_** [that **the boys_**PL**_** teased on the playground] **were** late for school.

Sometimes, featurally similar information in non-target positions intrudes on retrieval of the target, modulating acceptability and reading times. Such effects are commonly referred to as “similarity-based interference” (Gordon et al., 2001; Lewis and Vasishth, 2005; Lewis et al., 2006; Van Dyke and McElree, 2006; Van Dyke and Johns, 2012). The current study investigates the conditions under which such effects arise during retrieval for agreement processing.

Previous research on memory retrieval for dependency formation during real-time sentence comprehension has revealed a mixed profile of successes and failures with respect to interference effects. Some dependencies, like those involving subject-verb agreement, negative polarity item licensing, case licensing, and ellipsis, are highly susceptible to interference (Clifton et al., 1999; Pearlmutter et al., 1999; Vasishth et al., 2008; Wagers et al., 2009; Xiang et al., 2009, 2013; Martin et al., 2012; Dillon et al., 2013; Sloggett, 2013; Tanner et al., 2014; Lago et al., 2015; Tucker et al., 2015; Parker and Phillips, 2016). But other dependencies, like those involving reflexives, control, strong crossover binding, and bound variable pronouns, are more resistant to interference (Clifton et al., 1999; Dillon et al., 2013; Kush and Phillips, 2014; Kush et al., 2015, 2017), or require specific configurations for interference to obtain (Parker et al., 2015; Parker and Phillips, 2017).

The question of why different dependencies show different profiles with respect to interference remains unresolved (see Parker and Phillips, 2017, for discussion). However, many existing accounts agree that for the dependencies that do show interference, such as subject-verb agreement, interference reflects misretrieval of a feature-appropriate items from a structurally irrelevant position (Wagers et al., 2009; Dillon et al., 2013; Tanner et al., 2014; Lago et al., 2015; Tucker et al., 2015; Parker and Phillips, 2017; Tucker and Almeida, 2017). A key prediction of this retrieval-based account is that interference should generalize across a broad range of structural configurations, since the same error-prone retrieval mechanism should apply whenever a comprehender attempts agreement licensing (McElree, 2000; McElree et al., 2003; Lewis and Vasishth, 2005; Lewis et al., 2006).

However, recent research on retrieval for subject-verb thematic binding suggests that interference effects can also be modulated by the encoding mechanisms. For instance, Van Dyke and McElree tested sentences like those in (2). In both sentences, the critical verbs (*moaned* and *compromised*) require an animate subject, motivating the use of animacy as a retrieval cue for these dependencies (Van Dyke, 2007; Van Dyke and McElree, 2011). Despite similar retrieval requirements in (2a-b), Van Dyke and colleagues observed contrasting profiles: interference effects arose when a structurally-irrelevant animate distractor (in bold) occurred inside a prepositional phrase (PP), as in (2a), but not when it occurred as a direct object, as in (2b).

(2)
  (a) The pilot remembered that the lady who was sitting near ***the smelly man*** moaned about a friend.
  (b) The attorney who the judge realized had rejected ***the witness*** in the case compromised.

This contrast is surprising because it is not predicted by existing retrieval accounts (McElree, 2000, 2006; McElree et al., 2003; Lewis and Vasishth, 2005). Existing accounts predict similar interference profiles for (2a-b), since the same interference-prone mechanism is assumed to apply whenever the comprehender attempts retrieval for thematic binding.

Van Dyke and McElree (2011) argued that the source of the contrast in (2) is the syntactic encoding. Specifically, they suggested that PPs and direct objects differ in the distinctiveness of their memory representations based on an argument hierarchy, making them differentially susceptible to interference. Many grammatical theories make a hierarchical distinction between core thematic arguments (e.g., subjects, direct objects), which play a prominent role in establishing the meaning of the sentence, and modifying oblique arguments, including PPs, which possess little discriminating syntactic information (e.g., PPs lack a theta role) and play a less prominent role in building meaning (Keenan and Comrie, 1977; Chomsky, 1981; Frazier and Clifton, 1996; Van Valin and LaPolla, 1997; Bresnan, 2001; Culicover and Jackendoff, 2005). Drawing on this distinction, Van Dyke and McElree (2011) hypothesized that the prominent grammatical function of core arguments makes the syntactic aspects of their memory encoding more distinctive, relative to oblique arguments, and hence easier to reject or accept based on their match to the syntactic retrieval cues. On this view, the distinctiveness of the syntactic features of the direct object in (2b) produces a salient mismatch with the subject retrieval cues of the verb, making them relatively easier to rule out. Conversely, less distinctive representations, like the oblique PP in (2a), are not salient enough to produce a strong mismatch with the syntactic retrieval cues, and hence are more likely to interfere, yielding the contrast observed in (2). Crucially, unlike previous accounts of interference that place the blame on the retrieval mechanisms, Van Dyke and McElree (2011) suggested that in the case of thematic binding, it is the encoding mechanisms that mediate interference.

Additional evidence of interference based on the thematic-semantic properties of the distractor encoding comes from Cunnings and Sturt (2018). Cunnings and Sturt manipulated sentence plausibility as a diagnostic of interference in sentences like (3). In (3), the critical verb *shattered* triggers a retrieval to recover its direct object. They manipulated whether the retrieval target, the direct object of the matrix verb, e.g., *the plate/letter*, was a plausible direct object of the critical verb, as well as the plausibility of a distractor embedded inside an intervening PP, e.g., *the cup/tie*.

(3) Sue remembered the plate/letter that the butler with **the cup/tie** accidently shattered today in the dining room.

Cunnings and Sturt observed a significant main effect of plausibility, such that implausible sentences were read more slowly than plausible sentences at the critical verb and spillover regions. They also found that this effect was modulated by the plausibility of the distractor, such that the plausibility effect was attenuated in sentences with a plausible distractor, e.g., *the cup*. These findings support Van Dyke and McElree’s proposal that oblique arguments, such as PPs, trigger interference, and extend their findings by showing that retrieval for thematic binding is sensitive to a broader range of thematic-semantic properties of the distractor encoding beyond animacy, e.g., [+shatterable].

A concern for the encoding hypothesis proposed by Van Dyke and McElree (2011) is that not all core arguments are equally resistant to interference. For instance, although they found that distractors in a direct object position resist interference during retrieval for thematic binding, they also found that distractors in a subject position reliably triggered interference, despite being a core argument. Van Dyke and McElree (2011) suggested that interference from subject distractors is expected because they match the syntactic cues from the verb, and it is only when a core argument mismatches the syntactic cues, as in the case of a direct object distractor, that they are precluded from retrieval, resulting in an effect they called ‘syntactic gating.’

The finding that subject distractors trigger interference is also consistent with the recent proposal that the prominence of the distractor modulates interference (Cunnings and Felser, 2013; Engelmann et al., 2015; Patil et al., 2016). For instance, subjects are more prominent than direct objects in terms of their hierarchical position and discourse function, which makes them more salient in memory, and hence more likely to interfere at retrieval. On this view, argument status is but a single factor that determines susceptibility to interference.

The encoding hypothesis proposed by Van Dyke and McElree (2011) has important implications for our understanding of how we encode and navigate linguistic structures in memory. However, their proposal has never been explicitly tested, and the generality of the effects on which it is based remains unclear. Furthermore, the principle of argument status is based on both the syntactic and thematic-semantic properties of the constituent, and it remains unclear which of these properties is responsible for the observed contrast, making it difficult to distinguish the various accounts relating to argument status, cue-overlap (syntactic gating), and prominence. It is thus important to test whether the contrast observed in (2) generalizes to a broader set of structural environments and a wider range of linguistic dependencies to better understand what properties cause memory retrieval mechanisms to succeed and fail during sentence comprehension.

### The Present Study

The present study uses interference effects in the comprehension of subject-verb agreement (‘agreement attraction’) to test Van Dyke and McElree’s (2011) hypothesis that interference is mediated by the argument status of the distractor. Agreement attraction arises when a comprehender fails to notice that a plural-marked verb erroneously agrees with a distractor noun (termed an ‘attractor’) that is not its syntactic subject. It manifests as eased processing and boosted acceptability during agreement processing, relative to sentences that should be equally acceptable or unacceptable, resulting in an effect known as ‘agreement attraction.’ For instance, Wagers and colleagues used self-paced reading to examine the processing of grammatical and ungrammatical subject-verb agreement dependencies like those in (4). The sentence in (4b) is ungrammatical because the plural verb *were* does not agree in number with the head of its subject noun phrase (NP) *key*.

(4)
  (a) The key to *the cabinet(s)* unsurprisingly was rusty after years of disuse.
  (b) ^∗^The key to *the cabinet(s)* unsurprisingly were rusty after years of disuse.

Wagers and colleagues found that in grammatical sentences like (4a), the number marking on the plural attractor *cabinets* did not impact acceptability or reading times after the verb. However, in ungrammatical sentences like (4b), the plural attractor *cabinets*, which matched the number of the verb *were*, boosted acceptability and facilitated reading times after the verb, relative to the ungrammatical condition with the singular noun *cabinet*. Wagers and colleagues argued that the facilitation observed in sentences like (4b) was due to incorrect retrieval of the plural attractor, which matches the plural retrieval cue at the verb. According to this account, encountering the plural verb *were* triggers a retrieval process to recover a constituent in memory that matches the cues [+subject] and [+plural]. In sentences that give rise to agreement attraction, like (4b), the target subject is encoded as [+subject] and [-plural], whereas the attractor is encoded as [-subject] and [+plural]. In this scenario, the retrieval processes triggered at the verb may retrieve the ‘attractor’ based on the partial match to the [+plural] cue, leading to the false impression that agreement is licensed (see also Dillon et al., 2013; Tanner et al., 2014; Lago et al., 2015; Tucker et al., 2015; Parker and Phillips, 2017; Tucker and Almeida, 2017).

Agreement attraction is not simply a case of proximity concord (Quirk et al., 1985) or local coherence (Tabor et al., 2004), as attraction is observed when the attractor does not intervene between the verb and its subject, as shown in (5).

(5) ^∗^The *runner(s)* who the driver see each morning always wave.

Agreement attraction provides an ideal test of Van Dyke and McElree’s (2011) hypothesis that interference is mediated by the argument status of the distractor because susceptibility to attraction can be examined in a broad range of configurations, such as those with attractors in core and oblique argument positions. However, the vast majority of studies on agreement attraction have relied on a narrow range of configurations involving oblique PP attractors (see Hammerly et al., 2018, for a recent review), motivating further research. A small number of studies have reported evidence of attraction from constituents in core argument positions, such as matrix subjects like (5) (Clifton et al., 1999; Wagers et al., 2009) and direct objects embedded inside a relative clause (Dillon et al., 2013). But, there has not yet been a direct, side-by-side comparison of attractors in core argument and oblique positions for subject-verb agreement to evaluate Van Dyke and McElree’s (2011) proposal. Furthermore, existing studies employed different experimental designs, items, and methodologies, making it difficult to compare interference profiles across configurations (see Jäger et al., 2017, for a Bayesian meta-analysis of attraction effects in comprehension). These issues are addressed in the current study.

### Overview of Experiments

Three self-paced reading experiments were designed to test Van Dyke and McElree’s (2011) hypothesis that interference is mediated by the argument status of the distractor using an agreement attraction paradigm. Specifically, we used the amount of attraction generated by core argument vs. oblique argument attractors to diagnose the distinctiveness of the respective encodings. Experiment 1 directly compared oblique (PP) argument attractors and core argument (direct object) attractors embedded inside a subject-modifying relative clause, as shown in (6), and Experiment 2 compared two types of core argument attractors (subject and direct object) in configurations like (7). To preview, attraction effects were observed for oblique attractors (PP attractors), but the effect was nullified for core argument attractors (subject and direct object attractors). These results are consistent with Van Dyke and McElree’s (2011) proposal that interference is mediated by the argument status of the distractor, but challenge accounts that claim that subjects should produce more interference due to their prominence (cf. Engelmann et al., 2015).

(6)
  (a) **PP attractor**
    ^∗^The waitress who sat near the girls unsurprisingly were unhappy …
  (b) **Direct object attractor**
    ^∗^The waitress who sat the girls unsurprisingly were unhappy …

(7)
  (a) **Direct object attractor**
    ^∗^The celebrity who insulted the journalists certainly were upset …
  (b) **Subject attractor**
    ^∗^The celebrity who the journalists insulted certainly were upset …

Experiment 3 then tested core argument attractors in a syntactically oblique position (oblique agents), as shown in (8), to determine whether the lack of attraction for items in core argument positions is driven by their syntactic position or their thematic-semantic properties that jointly define their argument status.

(8) **Oblique agent attractor**
  ^∗^The house that had been built by the workers sadly were falling …

Results showed that oblique agents resist attraction, which suggests that the lack of attraction for core arguments is not driven by the attractor’s syntactic position, but rather its thematic-semantic properties. Taken together, the results of Experiments 1–3 provide converging evidence in favor of the proposal that interference effects are mediated by the argument status of the interfering item (Van Dyke and McElree, 2011), and motivate a more comprehensive account of agreement processing that must consider both encoding and retrieval mechanisms.

## Experiment 1: Direct Comparison of Core Vs. Oblique Arguments

Experiment 1 directly compared PP and direct object attractors using self-paced reading to test Van Dyke and McElree’s (2011) proposal that interference during retrieval for linguistic dependency formation is mediated by the argument status of the interfering item. According to their proposal, the encoding of oblique arguments, such as PPs, is less distinctive than that of core arguments like subjects and objects. On this view, the encoding of oblique arguments is not salient enough to trigger a mismatch to the syntactic cues at retrieval, making interfering items in oblique argument positions more likely to interfere at retrieval. If agreement attraction, as a specific kind of interference, is mediated by the argument status of the attractor, then we expect to find a substantially reduced or nullified attraction effect for sentences with a core argument direct object attractor, relative to sentences with an oblique argument PP attractor. However, if argument status does not mediate attraction, then we expect comparable attraction effects for PP and direct object attractors.

Based on previous studies of agreement attraction in comprehension (e.g., Wagers et al., 2009; Dillon et al., 2013), attraction is predicted to manifest as a reduced reading time disruption for ungrammatical sentences with a plural attractor, relative to ungrammatical counterparts with a singular attractor. By contrast, the absence of an attraction effect is predicted to appear as disrupted reading times for ungrammatical sentences, with no statistically significant difference in reading times between the ungrammatical sentences.

### Participants

Participants were 60 native speakers of English who were recruited using Amazon’s Mechanical Turk web service^1^. All participants in this and the following experiments provided informed consent and were screened for native speaker abilities. The screening probed knowledge of the constraints on English tense, modality, morphology, ellipsis, and syntactic islands. Participants were compensated $4.00. The experiment lasted approximately 25 min.

### Materials

Experimental materials consisted of 48 sets of 8 items like those shown in **Table 1**. Three experimental factors were manipulated, including grammaticality (grammatical vs. ungrammatical), attractor number (singular vs. plural), and attractor argument status (direct object vs. PP). In all conditions, the target subject was modified by a subject relative clause that contained the attractor, followed by the main clause verb phrase, which consisted of the critical agreeing auxiliary verb and a 4–7 word spillover region. The target subject was always singular. The relative clause verb never overtly expressed agreement to prevent attraction before the critical region. Grammaticality was manipulated by varying the number feature of the critical agreeing verb (grammatical conditions = *was*, ungrammatical conditions = *were*). Attractor number was manipulated by varying the number of the attractor, such that it appeared in either singular or plural form. Based on previous studies on agreement attraction in comprehension, such as Wagers et al. (2009) and Dillon et al. (2013), singular attractors were predicted to cause no attraction, whereas plural embedded attractors were potential sources of attraction, but only in the ungrammatical conditions, where the target subject and critical verb mismatched in number. Attractor argument status was manipulated by varying the position of the attractor, such that it appeared in either direct object or PP position immediately following the relative clause verb. Lexical items were chosen to create maximally similar sentences for direct object and PP attractor conditions. Crucially, the linear distance between the attractors and critical agreeing verbs was identical in each configuration to prevent biases due to differences in recency, decay, or passive memory dynamics unrelated to the processing of subject-verb agreement (e.g., Van Dyke and Lewis, 2003). The full set of experimental materials can be found in the **Supplementary Materials**.

**Table 1:** Sample set of items for Experiment 1.

**Direct object attractor**
*Grammatical, PL attractor*
The waitress who sat the girls unsurprisingly was unhappy about all the noise.
*Grammatical, SG attractor*
The waitress who sat the girl unsurprisingly was unhappy about all the noise.
*Ungrammatical, PL attractor*
The waitress who sat the girls unsurprisingly were unhappy about all the noise.
*Ungrammatical, SG attractor*
The waitress who sat the girl unsurprisingly were unhappy about all the noise.
**PP attractor**
*Grammatical, PL attractor*
The waitress who sat near the girls unsurprisingly was unhappy about all the noise.
*Grammatical, SG attractor*
The waitress who sat near the girl unsurprisingly was unhappy about all the noise.
*Ungrammatical, PL attractor*
The waitress who sat near the girls unsurprisingly were unhappy about all the noise.
*Ungrammatical, SG attractor*
The waitress who sat near the girl unsurprisingly were unhappy about all the noise.

*SG, singular; PL, plural.*

The 48 target items were distributed across 8 lists in a Latin square design and combined with 96 grammatical filler sentences of similar length and complexity, such that each participant read a total of 144 sentences. All sentences were followed by a ‘yes/no’ comprehension question that addressed various parts of the sentence to prevent participants from developing superficial reading strategies that would allow them to answer the question without reading the entire sentence.

### Procedure

The experiment was conducted using the online experiment platform Ibex (Drummond, 2018), which allows self-paced reading experiments to be deployed in a standard web browser. Sentences were initially masked by dashes, with white spaces and punctuation intact. Participants pushed the space bar to reveal each word. Presentation was non-cumulative, such that the previous word was replaced with dashes when the next word appeared. On-screen feedback was provided for incorrect answers to the comprehension questions. The order of presentation was randomized for each participant. To ensure that participants completed the task as directed, an instructional manipulation check was used (Oppenheimer et al., 2009). Instructional manipulation checks ensure that participants are completing the task as directed by asking them to ignore the standard response format and provide a confirmation that they have read the instructions.

### Analysis

Only participants with at least 80% accuracy on comprehension questions were used in the analysis. Two participants were removed for performance below 80%. Four regions of interest were identified: the word immediately preceding the critical agreeing verb (pre-critical region), the agreeing verb (critical region), and the two words immediately following the verb (spillover regions 1 and 2, respectively). Based on previous studies that tested agreement attraction using self-paced reading, attraction effects were predicted to manifest starting at the regions immediately following the critical verb, e.g., spillover regions 1 and 2. Statistical analyses were carried out with linear mixed-effects models using the *lmerTest* package (Kuznetsova et al., 2014) in the *R* software environment (R Development Core Team, 2018). Analyses were carried out over the raw, untrimmed data, since recent research on attraction suggests that data transformations, such as those involving log-transformation or outlier removal (trimming), can obscure attraction effects (Staub, 2010; Lago et al., 2015; Tucker and Almeida, 2017; Villata et al., 2018).^2^ Models were defined using orthogonal contrast coding to examine the effects of grammaticality, attractor number, and their interaction (grammaticality × attractor number) for each region of interest. Following Dillon et al. (2013), additional models were defined to focus on the effect of attraction (i.e., the amount of facilitation for ungrammatical sentences with a plural attractor relative to ungrammatical sentences with a singular attractor), labeled as ‘attraction’ in the coefficient tables, and the interaction of attraction with attractor argument status to determine whether PP and object attractors were differentially susceptible to attraction. All models were fit with a full variance-covariance matrix, i.e., a maximal random effects structure, with random intercepts and slopes for all fixed effect predictors by participants and items (Barr et al., 2013). If there was a convergence failure, or if the model converged but the correlation estimates were high, the random effects structure was simplified. A fixed effect was considered significant if its absolute *t-*value was greater than 2, which indicates that its 95% confidence interval did not include 0 (Gelman and Hill, 2007).

### Results

**FIGURE 1:**
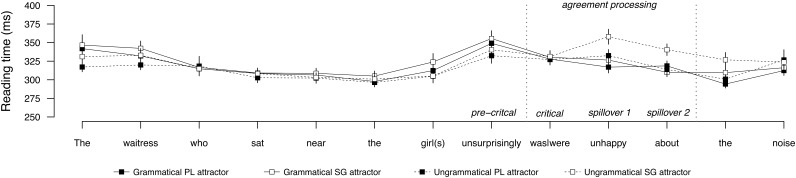
Word-by-word reading times for the PP attractor conditions in Experiment 1. Error bars indicate standard error of the mean.

**FIGURE 2:**
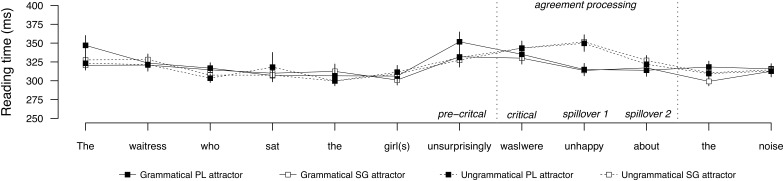
Word-by-word reading times for the direct object attractor conditions in Experiment 1. Error bars indicate standard error of the mean.

**Figure 1** shows the average word-by-word reading times for sentences with a PP attractor, and **Figure 2** shows the same for sentences with a direct object attractor. Mean reading times by condition at the regions of interest are provided in **Table 2**, and the results of the statistical analyses are reported in **Table 3**. Contrasting profiles were observed for prepositional and direct object attractors. In the PP attractor conditions, no effects were observed in the pre-critical or critical regions. As expected, the following spillover regions showed a main effect of grammaticality (Spillover 1 and 2), a main effect of attractor number (Spillover 1), a significant effect of attraction (Spillover 1 and 2), and a significant interaction between grammaticality and attractor number (Spillover 2). In these regions, ungrammatical sentences were read more slowly than grammatical sentences, but this processing disruption was nullified for ungrammatical sentences with a plural attractor, relative to ungrammatical sentences with a singular attractor. This pattern reflects the behavioral signature of agreement attraction, replicating previous results (e.g., Wagers et al., 2009; Dillon et al., 2013; Lago et al., 2015; Tucker et al., 2015; Parker and Phillips, 2017; Tucker and Almeida, 2017).

**Table 2:** Mean reading times (ms) by condition at the regions of interest for Experiment 1.

	Regions			
	Pre-critical	Critical	Spillover 1	Spillover 2
**PP attractor**				
Grammatical, PL attractor	349	327	317	318
Grammatical, SG attractor	355	330	326	310
Ungrammatical, PL attractor	332	327	332	313
Ungrammatical, SG attractor	340	331	357	340
**Direct object attractor**				
Grammatical, PL attractor	331	330	313	317
Grammatical, SG attractor	351	335	314	313
Ungrammatical, PL attractor	327	343	351	326
Ungrammatical, SG attractor	330	343	349	321

**Table 3:** Summary of statistical analyses for PP attractor conditions and direct object attractor conditions in Experiment 1.

	Regions											
	Pre-critical			Critical			Spillover 1			Spillover 2		
	$\hat{\text{β}}$	*SE*	*t*	$\hat{\text{β}}$	*SE*	*t*	$\hat{\text{β}}$	*SE*	*t*	$\hat{\text{β}}$	*SE*	*t*
**PP attractor**												
Grammaticality	-7.94	4.61	-1.72	0.09	2.87	0.03	**11.70**	**3.33**	**3.50**	**6.36**	**2.52**	**2.51**
Attractor number	3.64	4.49	0.81	1.64	2.87	0.57	**8.73**	**3.33**	**2.61**	4.53	2.52	1.79
Grammaticality × attractor number	0.38	4.49	0.08	0.44	2.87	0.15	4.00	3.33	1.21	**8.77**	**2.52**	**3.47**
Attraction	4.06	6.45	0.63	1.99	4.53	0.44	**12.72**	**4.99**	**2.54**	**13.27**	**4.47**	**2.96**
**Direct object attractor**												
Grammaticality	-6.35	4.53	-1.40	5.41	3.44	1.57	**18.23**	**3.99**	**4.56**	4.45	3.85	1.15
Attractor number	-5.96	4.53	-1.31	-1.25	3.44	-0.36	0.14	4.80	0.03	2.04	3.19	0.64
Grammaticality × attractor number	4.0	4.53	0.88	1.32	3.44	0.38	0.78	3.52	0.22	0.47	2.77	0.17
Attraction	-1.87	6.49	-0.28	0.11	5.53	0.02	0.94	7.42	0.12	2.53	3.89	0.65
Attraction × argument status	5.87	8.53	0.68	1.77	6.53	0.27	11.56	7.74	1.49	**10.73**	**5.26**	**2.04**

*Significant coefficients (|t| > 2) are in bold.*

In the direct object attractor conditions, no effects were observed in the pre-critical or critical regions. The following spillover region showed a main effect of grammaticality (Spillover 1), carried by longer reading times in the ungrammatical sentences relative to grammatical sentences. In contrast to the PP attractor conditions, there was no evidence of attraction in any region, as reading times between ungrammatical conditions did not diverge.

The contrast between PP and direct object attractors with respect to attraction was supported by a significant interaction between attraction and attractor argument status, carried by the significant attraction effect for the PP attractor conditions.

### Discussion

Experiment 1 directly compared PP and direct object attractors to test Van Dyke and McElree’s (2011) proposal that interference effects are mediated by the argument status of the interfering item. This proposal claims that core arguments, such as direct objects, are encoded in memory more distinctly than oblique arguments, such as PPs, making them easier to reject when they mismatch the retrieval cues, and hence less likely to interfere at retrieval. Experiment 1 revealed that oblique arguments in PP position interfered during retrieval for agreement processing, yielding a clear agreement attraction effect, but core arguments in direct object position did not. These results are closely aligned with Van Dyke and McElree’s (2011) proposal, and extend their findings by showing that the contrast between core and oblique arguments with respect to interference extends to a wider range of dependencies such as subject-verb agreement.

A concern with the results of Experiment 1 is that the critical interactions of grammaticality × attractor number and attraction × attractor argument status were observed two words after the critical verb. There are two reasons why we might see these effects appear after the critical word. First, recent work on the timing of agreement attraction effects suggests that attraction is an error-driven process that manifests in the late stages of agreement processing (Lago et al., 2015; Parker and Phillips, 2017). The observation of a late interaction is consistent with this view. Second, observing an effect one or two regions downstream from the critical region is expected in self-paced reading tasks, since participants often adopt a fixed rhythm in advancing through the sentence (Witzel et al., 2012).

Another concern with Experiment 1 is that it failed to replicate attraction in configurations that have been shown to yield attraction in previous studies. For instance, Dillon et al. (2013) observed attraction when the attractor appeared as the direct object of a subject-modifying relative clause. This configuration is nearly identical to the direct object attraction condition tested in Experiment 1, which did not show attraction. One possibility is that the lack of attraction for direct object attractors in Experiment 1 is due to a lack of statistical power. There are three reasons why the current results are unlikely to reflect low power. First, we observed a positive attraction effect in maximally similar sentences involving PP attractors, which suggests that there was sufficient power to elicit attraction. Second, Experiment 1 had more power than previous studies that elicited attraction. For instance, Experiment 1 relied on 6 observations per condition, with 60 participants, yielding a total of 360 points for analysis. By comparison, Dillon et al. (2013) elicited attraction with less power (6 observations per condition, with 40 participants, for a total of 240 data points). Other studies that used self-paced reading to elicit attraction are similarly patterned. For example, Wagers et al. (2009) elicited attraction using self-paced reading in a design with exactly half the power of Experiment 1 in the current study. Thus, the lack of attraction under superficially similar conditions in the present study is unlikely to reflect an issue of statistical power. Third, we conducted a *post hoc* power analysis using the *simr* package in R over the final linear-mixed-effects model (including the main effects and their interaction) at the second spillover region, which showed the critical interaction between attraction and attractor position. According to this analysis, the observed power was at 74%, which suggests that lack of power is an unlikely cause for the contrast. However, since the standard recommendation is that the target power rate should be at least 80% (Cohen, 1962, 1988, 1992), the issue of power is addressed further in Experiment 2.

Another possibility is that the contrast between the current study and Dillon et al. (2013) reflects variability in the materials used by Dillon and colleagues. In their study, Dillon et al. (2013) reported the use of direct object attractors in their sample set of materials (see **Table 1** of their study). However, their full materials list shows that they used a combination of direct object and PP attractors exactly of the form tested in Experiment 1, with nearly 40% of their items using PP attractor configurations. It is possible that the attraction effects that they observed were driven by the PP conditions, in which case our studies pattern similarly with respect to attraction effects for prepositional and direct object attractors.

A third issue with Experiment 1 concerns the relationship between encoding accounts of interference (e.g., Van Dyke and McElree, 2011) and accounts of prominence and cue-matching (e.g., Engelmann et al., 2015). According to the encoding account, core arguments, like direct object attractors, are encoded in memory more distinctly than oblique arguments, like PP attractors, and hence, are less likely to interfere. The strong view of this proposal would be that all core arguments, including direct objects and subjects, should resist interference, by virtue of their argument status, regardless of their syntactic position. However, recent accounts of prominence and cue-matching (e.g., Cunnings and Felser, 2013; Engelmann et al., 2015; Patil et al., 2016) predict divergent interference profiles for core arguments. Specifically, core arguments are predicted to trigger interference if they more closely match the retrieval cues from the verb, such as the subject cue, or are in a more prominent position in the sentence, such as in a subject position. As a result, subjects are predicted to be more likely to interfere at retrieval than items in less prominent positions, like direct objects, due to their heightened activation in memory (see Engelmann et al., 2015, for predictions from computational simulations). This possibility is tested in Experiment 2.

## Experiment 2: Direct Comparison of Subject and Object Attractors

Experiment 1 showed that a core argument in direct object position did not trigger agreement attraction. It is possible that an attractor in subject position, despite its status as a core argument, might trigger attraction because it is highly accessible, both in terms of its match to the subject retrieval cue of the verb (Van Dyke and McElree, 2011) and its grammatical prominence (Engelmann et al., 2015). To test this hypothesis, Experiment 2 directly compared subject and direct object attractors in maximally similar configurations like those shown in (9) using self-paced reading.

(9)
  (a) **Direct object attractor**
    ^∗^The celebrity who insulted the journalists certainly were upset …
  (b) **Subject attractor**
    ^∗^The celebrity who the journalists insulted certainly were upset …

If interference effects are mediated by the match to the retrieval cues or prominence, then we expect contrasting profiles for subject and object attractors, with stronger attraction effects predicted for subject attractors, since they provide a better match to the retrieval cues and are in a more prominent position. However, if interference effects are mediated by the argument status of the interfering item, as previously claimed (Van Dyke and McElree, 2011), then subject and object attractors should show similar profiles with respect to attraction because they share the same status as core arguments.

### Participants

Participants were 120 native speakers of English who were recruited using Amazon’s Mechanical Turk web service. This large sample size was chosen to increase statistical power (Vasishth and Nicenboim, 2016) to address the concern that the lack of attraction for core arguments in Experiment 1 was due to low power. Participants were compensated $4.00. The experiment lasted approximately 25 min.

### Materials

Forty-eight item sets of the form shown in **Table 4** were constructed. The structure of the items followed the structure of the items used in Experiment 1, but held constant the argument status of the attractors, and instead manipulated their syntactic position. Attractors appeared either as the direct object of the relative clause verb, as in Experiment 1, or as the subject of the relative clause verb.

**Table 4:** Sample set of items for Experiment 2.

**Direct object attractor**
*Grammatical, PL attractor*
The celebrity who insulted the journalists certainly was upset about the claims.
*Grammatical, SG attractor*
The celebrity who insulted the journalist certainly was upset about the claims.
*Ungrammatical, PL attractor*
The celebrity who insulted the journalists certainly were upset about the claims.
*Ungrammatical, SG attractor*
The celebrity who insulted the journalist certainly were upset about the claims.
**Subject attractor**
*Grammatical, PL attractor*
The celebrity who the journalists insulted certainly was upset about the claims.
*Grammatical, SG attractor*
The celebrity who the journalist insulted certainly was upset about the claims.
*Ungrammatical, PL attractor*
The celebrity who the journalists insulted certainly were upset about the claims.
*Ungrammatical, SG attractor*
The celebrity who the journalist insulted certainly were upset about the claims.

*SG, singular; PL, plural.*

The 48 target items were distributed across 8 lists in a Latin square design and combined with the same 96 grammatical filler sentences from Experiment 1, such that each participant read a total of 144 sentences. All sentences were followed by a ‘yes/no’ comprehension question.

### Procedure and Analysis

Experiment 2 used self-paced reading, following the same procedure and analysis methods used in Experiment 1. Three participants were removed for failing the instructional manipulation check, and an additional 14 participants were removed for performance below 80%, leaving a total of 103 participants for data analysis.

### Results

**FIGURE 3:**
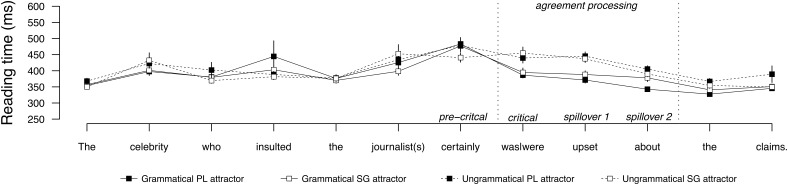
Word-by-word reading times for the direct object attractor conditions in Experiment 2. Error bars indicate standard error of the mean.

**FIGURE 4:**
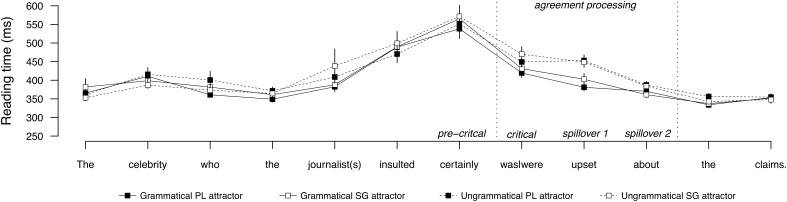
Word-by-word reading times for the subject attractor conditions in Experiment 2. Error bars indicate standard error of the mean.

**Figure 3** shows the average word-by-word reading times for sentences with a direct object attractor, and **Figure 4** shows the same for sentences with a subject attractor. Mean reading times by condition at the regions of interest are provided in **Table 5**, and the results of the statistical analyses are reported in **Table 6**. No effects were observed in the pre-critical conditions for either subject or object attractor conditions. Both subject and object attractor conditions showed a main effect of grammaticality at the critical verb region, which persisted to the second spillover region. There were no effects of attractor number or attraction in any region for subject and object attractor conditions. The second spillover region for object attractors showed a significant interaction between grammaticality and attractor number. Pairwise comparisons revealed that this interaction was carried by divergent reading times in the grammatical conditions, as grammatical sentences with a singular attractor were read more slowly than grammatical sentences with a plural attractor, relative to the ungrammatical conditions, which did not diverge. No other effects or interactions were observed.

**Table 5:** Mean reading times (ms) by condition at the regions of interest for Experiment 2.

	Regions			
	Pre-critical	Critical	Spillover 1	Spillover 2
**Direct object attractor**				
Grammatical, PL attractor	483	386	371	342
Grammatical, SG attractor	476	394	388	378
Ungrammatical, PL attractor	478	439	445	405
Ungrammatical, SG attractor	440	454	437	390
**Subject attractor**				
Grammatical, PL attractor	537	419	381	370
Grammatical, SG attractor	565	431	402	361
Ungrammatical, PL attractor	551	449	452	387
Ungrammatical, SG attractor	571	470	448	384

**Table 6:** Summary of statistical analyses for direct object attractor conditions and subject attractor conditions in Experiment 2.

	Regions											
	Pre-critical			Critical			Spillover 1			Spillover 2		
	$\hat{\text{β}}$	*SE*	*t*	$\hat{\text{β}}$	*SE*	*t*	$\hat{\text{β}}$	*SE*	*t*	$\hat{\text{β}}$	*SE*	*t*
**Direct object attractor**												
Grammaticality	-10.23	6.94	-1.47	**28.46**	**9.17**	**3.10**	**30.80**	**4.7**	**6.88**	**18.64**	**4.34**	**4.29**
Attractor number	-10.92	6.94	-1.57	5.48	7.80	0.70	2.50	4.48	0.55	5.027	3.92	1.28
Grammaticality × attractor number	-7.82	6.94	-1.12	1.51	7.93	0.19	-5.98	5.0	-1.17	-**12.65**	**4.16**	-**3.03**
Attraction	-18.86	12.71	-1.48	7.15	12.43	0.57	-3.95	6.89	-0.57	-7.66	5.20	-1.47
**Subject attractor**												
Grammaticality	1.45	12.03	0.12	**17.12**	**7.25**	**2.36**	**19.48**	**6.94**	**4.24**	**9.97**	**3.62**	**2.74**
Attractor number	15.43	12.05	1.28	2.46	7.25	0.33	4.34	5.74	0.75	-3.07	3.63	-0.84
Grammaticality × attractor number	-4.98	12.0	-0.41	8.73	7.25	1.20	-6.47	7.14	-0.90	1.37	3.76	0.36
Attraction	10.62	17.54	0.60	11.70	10.58	1.10	-2.15	8.33	-0.25	-1.78	5.25	-0.33
Attraction × argument status	28.98	18.14	1.59	4.97	16.12	0.30	1.82	10.93	0.16	5.96	7.34	0.81

*Significant coefficients (| t| > 2) are in bold.*

### Discussion

Experiment 2 compared subject and direct object attractors to test the hypothesis that a subject attractor should trigger attraction because it more closely matches the retrieval cues of the verb and is in a grammatically prominent position. Experiment 2 revealed two main findings. First, the prediction that subjects should trigger attraction was not supported by the reading time data from Experiment 2. Both subject and object attractor conditions showed a main effect of grammaticality, indicating that comprehenders were sensitive to the feature match between the verb and the target subject, but no evidence of attraction was found in any region from either subject or direct attractors. Second, Experiment 2 replicated the results of Experiment 1 by showing that direct objects resist attraction. This effect is notable given the high statistical power. This finding suggests that the lack of attraction for the direct object attractors in Experiment 1 cannot be reduced to low power. Taken together, the results of Experiments 1 and 2 are consistent with Van Dyke and McElree’s (2011) proposal that interference is mediated by the argument status of the distractor, but challenge the recent proposal that subjects are more likely to interfere due to their prominence (cf. Engelmann et al., 2015).

A concern with Experiment 2 is the interaction between grammaticality and attractor number for the direct object attractors at the second spillover region. This effect was carried by divergent reading times in the grammatical conditions, as grammatical sentences with a singular attractor were read more slowly than grammatical sentences with a plural attractor, relative to the ungrammatical conditions, which did not diverge. This effect is unexpected under accounts that assume that attraction is an error-driven process that is triggered only when the verb form violates the number prediction made by the subject (Wagers et al., 2009; see also, Lago et al., 2015; Parker and Phillips, 2017). According to this account, retrieval is not engaged in the grammatical conditions because the prediction is satisfied. The alternative view is that retrieval always occurs at the verb, regardless of grammaticality. However, the fact that the same effect was not observed in the subject attractor conditions or in Experiment 1 suggests that this effect may reflect a Type I error.

Another concern is that Experiment 2 failed to replicate Van Dyke and McElree’s (2011) syntactic gating effect, in which items in a subject position interfere at retrieval due to their match to the subject retrieval cues of the verb. There are two possibilities for why we might expect this difference. One possibility is that we tested a different dependency. We tested subject-verb agreement, which is a morpho-syntactic feature-matching process, whereas Van Dyke and McElree (2011) tested thematic binding, which is an interpretive process that aids in establishing the meaning of the sentence. Both processes require retrieval of the local subject at the verb, but they have different grammatical functions. It is thus not unreasonable to assume that they might use different cues to guide retrieval based on their different grammatical requirements. For instance, agreement might rely more on morpho-syntactic cues like person and number, whereas thematic binding might rely more on thematic-semantic cues, like animacy. However, it remains unclear why retrieval mechanisms would use different cues to target the same position.

A more likely possibility is that the contrasting profiles for subject distractors reflect differences in feature similarity between the target and distractor NPs in memory. In the items tested by Van Dyke and McElree (2011), both the subject distractor and target overlapped substantially with the retrieval cues (both were animate subjects), which can reduce the distinctiveness of the target and increase the opportunity for interference at retrieval (Watkins and Watkins, 1975; Nairne, 1988, 1990; Anderson and Lebiere, 1998; Anderson et al., 2004; McElree, 2006). By contrast, the subject attractor and target in Experiment 2 of the current study were more distinct in feature content (plural vs. singular), increasing their distinctiveness at retrieval, reducing the chances of interference from cue-overlap. Crucially, this account is consistent with Van Dyke and McElree’s (2011) general claim that interference is dependent on the *distinctiveness* of the information in memory. This account is also consistent with the recent proposal that interference depends on the degree to which the target and distractor match the retrieval cues (Parker and Phillips, 2017).

A more fundamental concern is that it is unclear why subject and object attractors differ from PP attractors with respect to interference. The results are consistent with Van Dyke and McElree’s (2011) proposal that interference (measured here in terms of attraction) is mediated by the argument status of the interfering item. But an item’s argument status is defined by both its syntactic and thematic-semantic properties. At this point, it is not clear which of these properties drives the contrasts observed in Experiments 1 and 2. This issue is addressed in Experiment 3.

## Experiment 3

Experiments 1 and 2 revealed that PP attractors differ from subject and object attractors with respect to agreement attraction. However, the source of this contrast remains unclear. On the one hand, the contrast could reflect the thematic-semantic status of the attractor, as originally hypothesized (e.g., Van Dyke and McElree, 2011). On the other hand, the contrast could simply reflect the attractor’s syntactic position. To distinguish these alternatives, Experiment 3 probed for attraction using core argument attractors that appeared in a PP position. Specifically, Experiment 3 tested configurations with an “oblique agent” attractor, where the attractor is a core thematic subject that appeared in a passive PP *by*-phrase (see **Table 5** for an example). If core arguments resist attraction by virtue of their thematic-semantic properties, then changes in their syntactic position should not impact their susceptibility to attraction. On this view, the oblique agent attractor should pattern with the core arguments from Experiments 1–2 (direct objects and subjects) by resisting attraction. However, if the contrast between core and oblique arguments is a consequence of their syntactic position, then the oblique agent should pattern with the PP attractor from Experiment 1 by triggering attraction.

### Participants

Participants were 120 native speakers of English who were recruited using Amazon’s Mechanical Turk web service. Participants were compensated $4.00. The experiment lasted approximately 25 min.

### Materials

Twenty-four item sets of the form shown in **Table 7** were constructed. Two factors were manipulated, grammaticality and attractor number. Across all conditions, the target subject was modified by a passivized relative clause that contained the attractor in a prepositional *by*-phrase (oblique agent), followed by the main clause VP and spillover regions. The passivized relative clause verb never overtly expressed agreement to prevent spurious interference effects prior to the critical verb.

**Table 7:** Sample set of items for Experiment 3.

*Grammatical, PL attractor*
The house that had been built by the workers sadly was falling into great disrepair.
*Grammatical, SG attractor*
The house that had been built by the worker sadly was falling into great disrepair.
*Ungrammatical, PL attractor*
The house that had been built by the workers sadly were falling into great disrepair.
*Ungrammatical, SG attractor*
The house that had been built by the worker sadly were falling into great disrepair.

*SG, singular; PL, plural.*

The 24 target items were distributed across 4 lists in a Latin square design and combined with the 48 grammatical filler sentences from Experiments 1–2, such that each participant read a total of 72 sentences. All sentences were followed by a ‘yes/no’ comprehension question.

### Procedure and Analysis

Experiment 3 used self-paced reading, following the same procedure and analysis methods used in Experiments 1 and 2. Two participants were removed for failing the instructional manipulation check, and an additional 11 participants were removed for performance below 80%, leaving a total of 107 participants for data analysis.

### Results

**FIGURE 5:**
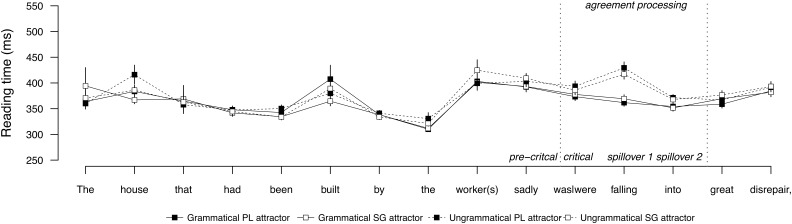
Word-by-word reading times for Experiment 3. Error bars indicate standard error of the mean.

**Figure 5** shows the average word-by-word reading times for Experiment 3. Mean reading times by condition at the regions of interest are provided in **Table 8**, and the results of the statistical analyses are reported in **Table 9**. No effects were observed in the pre-critical or critical regions. A main effect of grammaticality was observed in spillover regions 1 and 2. There was no evidence of attraction in any region.

**Table 8:** Mean reading times (ms) by condition at the regions of interest for Experiment 3.

	Regions			
	Pre-critical	Critical	Spillover 1	Spillover 2
Grammatical, PL attractor	392	373	361	354
Grammatical, SG attractor	393	377	369	351
Ungrammatical, PL attractor	403	393	429	371
Ungrammatical, SG attractor	409	386	416	367

**Table 9:** Summary of statistical analyses for Experiment 3.

	Regions											
	Pre-critical			Critical			Spillover 1			Spillover 2		
	$\hat{\text{β}}$	*SE*	*t*	$\hat{\text{β}}$	*SE*	*t*	$\hat{\text{β}}$	*SE*	*t*	$\hat{\text{β}}$	*SE*	*t*
Grammaticality	6.73	4.44	1.51	7.15	3.99	1.79	**28.86**	**4.30**	**6.70**	**8.22**	**3.04**	**2.70**
Attractor number	-1.87	4.44	-0.41	0.85	3.99	0.21	1.10	4.30	0.25	1.45	3.04	0.47
Grammaticality × attractor number	1.41	4.44	0.31	-3.04	3.99	-0.76	-4.91	4.3	-1.14	-0.13	3.04	-0.04
Attraction	-16.20	14.84	-1.09	3.76	5.96	0.63	5.95	7.90	0.75	1.62	3.60	0.45

*Significant coefficients (| t| > 2) are in bold.*

### Discussion

The goal of Experiment 3 was to determine whether the contrast between PP vs. subject and direct object attractors observed in Experiments 1 and 2 reflects the thematic-semantic status of the attractor or its syntactic position. This was achieved by testing oblique agents, which are core thematic subjects that appear in an oblique PP position. Results showed that oblique agents resist attraction, patterning with the core arguments from Experiments 1 and 2. These results suggest that the modulation of the attraction effect observed across Experiments 1 and 2 cannot be reduced to the syntactic position of the attractor, or at least a PP position. Instead, the currents results favor the proposal that core arguments resist interference by virtue of their thematic-semantic properties (e.g., Van Dyke and McElree, 2011).

A concern with the results of Experiment 3 is that they appear to conflict with previous studies on attraction in production, which have shown that the syntactic position of the attractor modulates attraction. For instance, attractors that are syntactically similar to agreement controllers (e.g., they c-command the verb) lead to more attraction errors than those that only precede the verb (Franck et al., 2006, 2010, 2015). The current results are not incompatible with these findings, and we do not deny that syntactic position plays an important role in attraction, at least in production. Rather, the results of Experiment 3 suggest that the current contrast between core and oblique arguments with regards to attraction in comprehension cannot be reduced to syntactic position. An important goal for future research is to determine whether the current contrasts observed in comprehension extend to agreement production.

## General Discussion

### Summary of Results

The goal of the current study was to test Van Dyke and McElree’s (2011) hypothesis that interference effects are mediated by the argument status of the distractor. This hypothesis states that core arguments, such as subjects and objects, are encoded more distinctly in memory than oblique arguments, such as PP objects, because core arguments play a more prominent role in establishing the meaning of the sentence, making them easier to accept or reject as retrieval candidates. The current study tested this hypothesis with an interference paradigm involving agreement attraction in three self-paced reading experiments. Experiment 1 directly compared PP and direct object attractors, and Experiment 2 directly compared direct object and subject attractors. Results showed a clear contrast: attraction was observed for PP attractors, but not for direct object or subject attractors. Experiment 3 then tested whether this contrast is a consequence of the syntactic or thematic-semantic properties of the attractors by testing core thematic arguments embedded in a PP (oblique agents). Results showed that oblique agents resisted attraction, patterning with the core arguments from Experiments 1 and 2. These results suggest that the contrast between core and oblique argument attractors is driven by their thematic-semantic properties, rather than their syntactic position.

**FIGURE 6:**
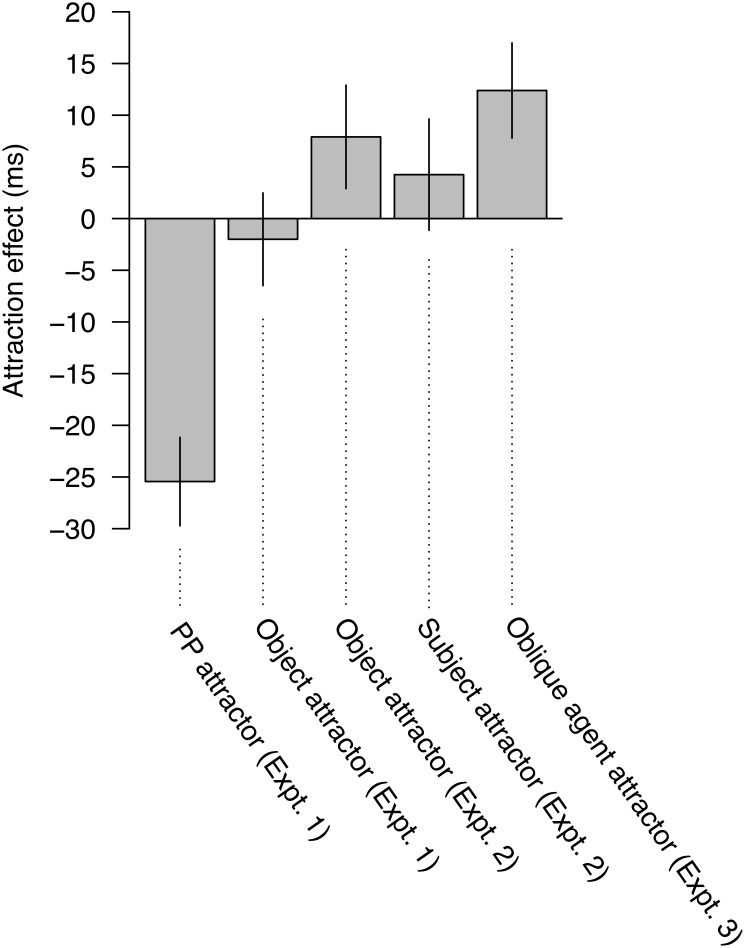
Comparison of the profiles observed in Experiments 1–3 for PP, subject, object, and oblique arguments. The attraction effect for each attractor position was calculated by subtracting the RTs for the ungrammatical plural attractor condition from the ungrammatical singular attractor condition in the first spillover region.

**Figure 6** provides a summary of the effects observed across each attractor position. This figure shows how PP attractors stand out, relative to subject, object, and oblique agent attractors, with respect to attraction effects. Taken together, the results of Experiments 1–3 provide converging evidence in favor of Van Dyke and McElree’s (2011) proposal that interference is mediated by the argument status of the interfering item. They also extend previous results by showing that such effects generalize to a broader set of syntactic contexts and a wider range of syntactic dependencies, such as subject-verb agreement, and clarify that it is specifically the thematic-semantic properties of the argument that mediate interference.

### Implications for Theories of Retrieval in Sentence Comprehension

The findings from the current study are unexpected under existing theories of memory retrieval in sentence comprehension, in the absence of a richer theory of memory representations and cues used in retrieval. Existing accounts, such as the prominent cue-based theory of memory retrieval, predict that interference effects for subject-verb agreement processing should generalize across syntactic contexts (Wagers et al., 2009; Dillon et al., 2013), based on the assumptions that the same interference-prone mechanism should apply whenever retrieval for agreement processing is required, and that interference is not mediated by the grammatical status of the attractor (McElree, 2000; McElree et al., 2003; Lewis and Vasishth, 2005). However, the current finding that agreement attraction is strongly modulated by the argument status of the attractor favors Van Dyke and McElree’s (2011) proposal that interference is mediated by the encoding of the interfering item, motivating a more comprehensive account of agreement processing that depends on both retrieval and encoding mechanisms.

The current results also suggest that the relationship between argument status and interference is more tightly connected than previously assumed. For instance, Van Dyke and McElree (2011) found that core arguments in subject position trigger interference for thematic binding. However, Experiment 2 of the current study found that interference from subject distractors does not extend to subject-verb agreement. As suggested earlier, the positive effect found by Van Dyke and McElree (2011) may reflect a multiple match effect, where both the distractor and target overlap in feature content, reducing the distinctiveness of the target. Controlling for this difference, the generalization that emerges from these studies is that interference is dependent on the distinctiveness of the interfering item according to an argument hierarchy.

More broadly, the current results suggest that the memory architecture for language processing is more grammatically sophisticated than previously assumed. In particular, the current results, taken together with the findings reported in Van Dyke and McElree (2011), suggest that memory encoding mechanisms are attuned to fine-grained distinctions relating to the argument hierarchies described in the formal literature (Keenan and Comrie, 1977; Chomsky, 1981; Frazier and Clifton, 1996; Van Valin and LaPolla, 1997; Bresnan, 2001; Culicover and Jackendoff, 2005). These features of the grammar are often overlooked in many prominent models of sentence processing, including models that rely on superficial heuristics, “good enough” representations, local coherence, and other surface statistics (e.g., Townsend and Bever, 2001; Ferreira et al., 2002; Tabor et al., 2004; Ferreira and Patson, 2007; Karimi and Ferreira, 2016). Specifically, the results of the current study imply that interference effects are rooted in grammatical principles, e.g., an argument hierarchy, motivating a theory of sentence comprehension in which the parser and grammar are more closely aligned than previously assumed (e.g., Townsend and Bever, 2001; Ferreira et al., 2002; Ferreira and Patson, 2007).

### Variability Across Studies

The current study showed that PP attractors trigger interference, but direct object and subject attractors do not. These results appear to be at odds with previous demonstrations of attraction that have used subject and object attractors. For instance, both Clifton et al. (1999) and Dillon et al. (2013) observed attraction from items that appeared in a direct object position, and Wagers et al. (2009) observed attraction from items in a matrix subject position. However, a closer examination of these contexts reveals critical differences that may explain why we see different profiles across studies.

For instance, a survey of the full materials list from Dillon et al. (2013) showed that a combination of both direct object and PP attractors was used in their study. It is possible that the attraction effects that they observed were triggered by the PP attractors, as shown in the current study. In Clifton et al. (1999) and Wagers et al. (2009), the attractors appeared in a subject or direct object position as the head of an object relative clause that contained the critical verb, e.g., *The musicians_PL_ who the reviewer_SG_ praise_PL_ …* or *Lucine dislikes the people_PL_ who the manager_SG_ think_PL_ …* In these configurations, the attractor must be retrieved at the verb anyway, independently of subject-verb agreement processing, to thematically bind the attractor as the object of the verb. It is possible that sensitivity to the number-matching attractor reflects the fact that multiple retrieval processes are triggered by the main agreeing verb, one of which targets the attractor. On this view, retrieval of the plural attractor as the object of the verb might give comprehenders the false impression that subject agreement is also licensed. No such effect is expected in the current study, as the critical verb always targeted the same item, namely the head noun of the main clause subject. A task for future research is to better understand how retrieval for agreement processing and thematic binding interact when they are triggered by the same verb.

### Extensions to Other Dependencies

Van Dyke and McElree (2011) showed that core and oblique arguments differ with respect to interference during retrieval for thematic binding, and the current study extends those results by showing that the contrast generalizes to subject-verb agreement dependencies. These results raise the question of whether other dependencies should show similar effects. The evidence thus far is inconclusive, warranting further research.

One dependency that is ripe for investigation involves reflexive licensing. The leading consensus is that retrieval for reflexive licensing resists interference from all non-target items (see Dillon, 2014, for a review), except in specific configurations when the target subject provides a particularly poor match to the retrieval cues (Parker and Phillips, 2017). The majority of the existing studies on retrieval for reflexive licensing have tested attractors that appeared as core arguments (e.g., subjects and direct objects). To the best of our knowledge, there has only been one study that tested whether oblique arguments trigger interference for reflexives. Andrews et al. (2016) tested sentences like *The motherly therapist(s) of the widow(s) eventually reassured themselves …* and found weak evidence of attraction from the number matching attractor *the widows* embedded in a PP. The findings from these studies provide some support for the current proposal that oblique argument attractors trigger interference, but core arguments do not. However, more systematic research is necessary to determine whether other dependencies pattern similarly. We leave investigation of this issue to future work.

## Conclusion

The current study showed that oblique arguments triggered interference during retrieval for subject-verb agreement processing, but core arguments did not. These results were presented as evidence that retrieval interference is dependent on the distinctiveness of the items in memory according to an argument hierarchy. These effects might be a general property of verbal dependencies, including subject-verb agreement and thematic binding (e.g., Van Dyke and McElree, 2011). Taken together, these results shed new light on the principles that govern the accessibility of information in working memory, and show that interference effects are informative not only about retrieval mechanisms, but also about the nature of the encoding mechanisms.

## Ethics Statement

The protocol was approved by the William & Mary Protection of Human Subjects Committee. All subjects gave written informed consent.

## Author Contributions

DP was responsible for the design and analysis of Experiments 1–2. DP and AA jointly designed and analyzed Experiment 3. The writing was done by DP, with contributions from AA.

## Conflict of Interest Statement

The authors declare that the research was conducted in the absence of any commercial or financial relationships that could be construed as a potential conflict of interest.

## Footnotes

1 https://aws.amazon.com/mturk

2 For comparison, analyses using the log-transformed reading time values are reported in the **Supplementary Materials**. The results are comparable, and the presence/absence of attraction does not differ between analysis methods.
