# Supplementary material for: Not All Phrases Are Equally Attractive: Experimental Evidence for Selective Agreement Attraction Effects
Source: Front Psychol. 2018 Aug 28;9:1566. doi: 10.3389/fpsyg.2018.01566 (PMC6121010; doi:10.3389/fpsyg.2018.01566)
Supplement: Supplementary file 1 [file Data_Sheet_1.PDF]

Supplementary Materials for "Not all phrases are equally attractive: Experimental evidence for selective agreement attraction effects" by Dan Parker and Adam An.

1. Experiment materials

1.1 Experiment 1 Materials

1	a	The waitress who sat the girls unsurprisingly was unhappy about all the noise.
1	b	The waitress who sat the girl unsurprisingly was unhappy about all the noise.
1	c	The waitress who sat the girls unsurprisingly were unhappy about all the noise.
1	d	The waitress who sat the girl unsurprisingly were unhappy about all the noise.
1	e	The waitress who sat near the girls unsurprisingly was unhappy about all the noise.
1	f	The waitress who sat near the girl unsurprisingly was unhappy about all the noise.
1	g	The waitress who sat near the girls unsurprisingly were unhappy about all the noise.
1	h	The waitress who sat near the girl unsurprisingly were unhappy about all the noise.
2	a	The dog that dug the holes unfortunately was covered in mud all over.
2	b	The dog that dug the hole unfortunately was covered in mud all over.
2	c	The dog that dug the holes unfortunately were covered in mud all over.
2	d	The dog that dug the hole unfortunately were covered in mud all over.
2	e	The dog that dug in the holes unfortunately was covered in mud all over.
2	f	The dog that dug in the hole unfortunately was covered in mud all over.
2	g	The dog that dug in the holes unfortunately were covered in mud all over.
2	h	The dog that dug in the hole unfortunately were covered in mud all over.
3	a	The boy who played the games apparently was more competitive than ever before.
3	b	The boy who played the game apparently was more competitive than ever before.
3	c	The boy who played the games apparently were more competitive than ever before.
3	d	The boy who played the game apparently were more competitive than ever before.
3	e	The boy who played in the games apparently was more competitive than ever before.
3	f	The boy who played in the game apparently was more competitive than ever before.
3	g	The boy who played in the games apparently were more competitive than ever before.
3	h	The boy who played in the game apparently were more competitive than ever before.
4	a	The mom who drove the kids unsurprisingly was tired of the bickering in the car.
4	b	The mom who drove the kid unsurprisingly was tired of all the bickering in the car.
4	c	The mom who drove the kids unsurprisingly were tired of all the bickering in the car.
4	d	The mom who drove the kid unsurprisingly were tired of all the bickering in the car.
4	e	The mom who drove with the kids unsurprisingly was tired of all the bickering in the car.

4	f	The mom who drove with the kid unsurprisingly was tired of the bickering in the car.
4	g	The mom who drove with the kids unsurprisingly were tired of all the bickering in the car.
4	h	The mom who drove with the kid unsurprisingly were tired of all the bickering in the car.
5	a	The kid who hid the toys definitely was in a lot of trouble.
5	b	The kid who hid the toy definitely was in a lot of trouble.
5	c	The kid who hid the toys definitely were in a lot of trouble.
5	d	The kid who hid the toy definitely were in a lot of trouble.
5	e	The kid who hid behind the toys definitely was in a lot of trouble.
5	f	The kid who hid behind the toy definitely was in a lot of trouble.
5	g	The kid who hid behind the toys definitely were in a lot of trouble.
5	h	The kid who hid behind the toy definitely were in a lot of trouble.
6	a	The policeman who patrolled the neighborhoods evidently was mentioned in the newspaper.
6	b	The policeman who patrolled the neighborhood evidently was mentioned in the newspaper.
6	c	The policeman who patrolled the neighborhoods evidently were mentioned in the newspaper.
6	d	The policeman who patrolled the neighborhood evidently were mentioned in the newspaper.
6	e	The policeman who patrolled through the neighborhoods evidently was mentioned in the newspaper.
6	f	The policeman who patrolled through the neighborhood evidently was mentioned in the newspaper.
6	g	The policeman who patrolled through the neighborhoods evidently were mentioned in the newspaper.
6	h	The policeman who patrolled through the neighborhood evidently were mentioned in the newspaper.
7	a	The goat who ate the cans evidently was seen on the side of the road.
7	b	The goat who ate the can evidently was seen on the side of the road.
7	c	The goat who ate the cans evidently were seen on the side of the road.
7	d	The goat who ate the can evidently were seen on the side of the road.
7	e	The goat who ate from the cans evidently was seen on the side of the road.
7	f	The goat who ate from the can evidently was seen on the side of the road.
7	g	The goat who ate from the cans evidently were seen on the side of the road.
7	h	The goat who ate from the can evidently were seen on the side of the road.
8	a	The scientist who prepared the experiments suddenly was scared about all the consequences.
8	b	The scientist who prepared the experiment suddenly was scared about all the consequences.
8	c	The scientist who prepared the experiments suddenly were scared about all the consequences.
8	d	The scientist who prepared the experiment suddenly were scared about all the consequences.
8	e	The scientist who prepared for the experiments suddenly was scared about all the consequences.
8	f	The scientist who prepared for the experiment suddenly was scared about all the consequences.
8	g	The scientist who prepared for the experiments suddenly were scared about all the consequences.
8	h	The scientist who prepared for the experiment suddenly were scared about all the consequences.
9	a	The student who wrote the professors likely was concerned about her low grade.

9	b	The student who wrote the professor likely was concerned about her low grade.
9	c	The student who wrote the professors likely were concerned about her low grade.
9	d	The student who wrote the professor likely were concerned about her low grade.
9	e	The student who wrote to the professors likely was concerned about her low grade.
9	f	The student who wrote to the professor likely was concerned about her low grade.
9	g	The student who wrote to the professors likely were concerned about her low grade.
9	h	The student who wrote to the professor likely were concerned about her low grade.
10	a	The journalist who read the reports apparently was investigated for fraudulent investigative journalism.
10	b	The journalist who read the report apparently was investigated for fraudulent investigative journalism.
10	c	The journalist who read the reports apparently were investigated for fraudulent investigative journalism.
10	d	The journalist who read the report apparently were investigated for fraudulent investigative journalism.
10	e	The journalist who read about the reports apparently was investigated for fraudulent investigative journalism.
10	f	The journalist who read about the report apparently was investigated for fraudulent investigative journalism.
10	g	The journalist who read about the reports apparently were investigated for fraudulent investigative journalism.
10	h	The journalist who read about the report apparently were investigated for fraudulent investigative journalism.
11	a	The boy who walked the dogs probably was scared about being bitten.
11	b	The boy who walked the dog probably was scared about being bitten.
11	c	The boy who walked the dogs probably were scared about being bitten.
11	d	The boy who walked the dog probably were scared about being bitten.
11	e	The boy who walked toward the dogs probably was scared about being bitten.
11	f	The boy who walked toward the dog probably was scared about being bitten.
11	g	The boy who walked toward the dogs probably were scared about being bitten.
11	h	The boy who walked toward the dog probably were scared about being bitten.
12	a	The woman who walked the dogs clearly was happy about the cross-country road trip.
12	b	The woman who walked the dog clearly was happy about the cross-country road trip.
12	c	The woman who walked the dogs clearly were happy about the cross-country road trip.
12	d	The woman who walked the dog clearly were happy about the cross-country road trip.
12	e	The woman who walked by the dogs clearly was happy about the cross-country road trip.
12	f	The woman who walked by the dog clearly was happy about the cross-country road trip.
12	g	The woman who walked by the dogs clearly were happy about the cross-country road trip.
12	h	The woman who walked by the dog clearly were happy about the cross-country road trip.
13	a	The nurse who called the doctors unsurprisingly was excited about the good news.
13	b	The nurse who called the doctor unsurprisingly was excited about the good news.
13	c	The nurse who called the doctors unsurprisingly were excited about the good news.
13	d	The nurse who called the doctor unsurprisingly were excited about the good news.
13	e	The nurse who called for the doctors unsurprisingly was excited about the good news.

13	f	The nurse who called for the doctor unsurprisingly was excited about the good news.
13	g	The nurse who called for the doctors unsurprisingly were excited about the good news.
13	h	The nurse who called for the doctor unsurprisingly were excited about the good news.
14	a	The director who called the assistants suddenly was intrigued by the secret report.
14	b	The director who called the assistant suddenly was intrigued by the secret report.
14	c	The director who called the assistants suddenly were intrigued by the secret report.
14	d	The director who called the assistant suddenly were intrigued by the secret report.
14	e	The director who called for the assistants suddenly was intrigued by the secret report.
14	f	The director who called for the assistant suddenly was intrigued by the secret report.
14	g	The director who called for the assistants suddenly were intrigued by the secret report.
14	h	The director who called for the assistant suddenly were intrigued by the secret report.
15	a	The artist who painted the houses certainly was impressed with the stunning view.
15	b	The artist who painted the house certainly was impressed with the stunning view.
15	c	The artist who painted the houses certainly were impressed with the stunning view.
15	d	The artist who painted the house certainly were impressed with the stunning view.
15	e	The artist who painted by the houses certainly was impressed with the stunning view.
15	f	The artist who painted by the house certainly was impressed with the stunning view.
15	g	The artist who painted by the houses certainly were impressed with the stunning view.
15	h	The artist who painted by the house certainly were impressed with the stunning view.
16	a	The director who called the actors certainly was surprised about the exciting news.
16	b	The director who called the actor certainly was surprised about the exciting news.
16	c	The director who called the actors certainly were surprised about the exciting news.
16	d	The director who called the actor certainly were surprised about the exciting news.
16	e	The director who called for the actors certainly was surprised about the exciting news.
16	f	The director who called for the actor certainly was surprised about the exciting news.
16	g	The director who called for the actors certainly were surprised about the exciting news.
16	h	The director who called for the actor certainly were surprised about the exciting news.
17	a	The thief who stole the stashes obviously was scared of being caught red-handed.
17	b	The thief who stole the stash obviously was scared of being caught red-handed.
17	c	The thief who stole the stashes obviously were scared of being caught red-handed.
17	d	The thief who stole the stash obviously were scared of being caught red-handed.
17	e	The thief who stole from the stashes obviously was scared of being caught red-handed.
17	f	The thief who stole from the stash obviously was scared of being caught red-handed.
17	g	The thief who stole from the stashes obviously were scared of being caught red-handed.
17	h	The thief who stole from the stash obviously were scared of being caught red-handed.
18	a	The author who read the books apparently was disappointed with the surprise ending.

18	b	The author who read the book apparently was disappointed with the surprise ending.
18	c	The author who read the books apparently were disappointed with the surprise ending.
18	d	The author who read the book apparently were disappointed with the surprise ending.
18	e	The author who read about the books apparently was disappointed with the surprise ending.
18	f	The author who read about the book apparently was disappointed with the surprise ending.
18	g	The author who read about the books apparently were disappointed with the surprise ending.
18	h	The author who read about the book apparently were disappointed with the surprise ending.
19	a	The solider who fought the terrorists likely was surrounded by a team of insurgents.
19	b	The solider who fought the terrorist likely was surrounded by a team of insurgents.
19	c	The solider who fought the terrorists likely were surrounded by a team of insurgents.
19	d	The solider who fought the terrorist likely were surrounded by a team of insurgents.
19	e	The solider who fought against the terrorists likely was surrounded by a team of insurgents.
19	f	The solider who fought against the terrorist likely was surrounded by a team of insurgents.
19	g	The solider who fought against the terrorists likely were surrounded by a team of insurgents.
19	h	The solider who fought against the terrorist likely were surrounded by a team of insurgents.
20	a	The millionaire who drove the cars allegedly was pulled over for drunk driving.
20	b	The millionaire who drove the car allegedly was pulled over for drunk driving.
20	c	The millionaire who drove the cars allegedly were pulled over for drunk driving.
20	d	The millionaire who drove the car allegedly were pulled over for drunk driving.
20	e	The millionaire who drove past the cars allegedly was pulled over for drunk driving.
20	f	The millionaire who drove past the car allegedly was pulled over for drunk driving.
20	g	The millionaire who drove past the cars allegedly were pulled over for drunk driving.
20	h	The millionaire who drove past the car allegedly were pulled over for drunk driving.
21	a	The librarian who read the books certainly was surprised by the abrupt conclusion.
21	b	The librarian who read the book certainly was surprised by the abrupt conclusion.
21	c	The librarian who read the books certainly were surprised by the abrupt conclusion.
21	d	The librarian who read the book certainly were surprised by the abrupt conclusion.
21	e	The librarian who read about the books certainly was surprised by the abrupt conclusion.
21	f	The librarian who read about the book certainly was surprised by the abrupt conclusion.
21	g	The librarian who read about the books certainly were surprised by the abrupt conclusion.
21	h	The librarian who read about the book certainly were surprised by the abrupt conclusion.
22	a	The chef who prepared the meals evidently was unhappy about the quality of the ingredients.
22	b	The chef who prepared the meal evidently was unhappy about the quality of the ingredients.
22	c	The chef who prepared the meals evidently were unhappy about the quality of the ingredients.
22	d	The chef who prepared the meal evidently were unhappy about the quality of the ingredients.
22	e	The chef who prepared for the meals evidently was unhappy about the quality of the ingredients.

22	f	The chef who prepared for the meal evidently was unhappy about the quality of the ingredients.
22	g	The chef who prepared for the meals evidently were unhappy about the quality of the ingredients.
22	h	The chef who prepared for the meal evidently were unhappy about the quality of the ingredients.
23	a	The gardener who pruned the hedges financially was unable to buy more plants.
23	b	The gardener who pruned the hedge financially was unable to buy more plants.
23	c	The gardener who pruned the hedges financially were unable to buy more plants.
23	d	The gardener who pruned the hedge financially were unable to buy more plants.
23	e	The gardener who pruned around the hedges financially was unable to buy more plants.
23	f	The gardener who pruned around the hedge financially was unable to buy more plants.
23	g	The gardener who pruned around the hedges financially were unable to buy more plants.
23	h	The gardener who pruned around the hedge financially were unable to buy more plants.
24	a	The athlete who kicked the referees obviously was disappointed about the bad call.
24	b	The athlete who kicked the referee obviously was disappointed about the bad call.
24	c	The athlete who kicked the referees obviously were disappointed about the bad call.
24	d	The athlete who kicked the referee obviously were disappointed about the bad call.
24	e	The athlete who kicked near the referees obviously was disappointed about the bad call.
24	f	The athlete who kicked near the referee obviously was disappointed about the bad call.
24	g	The athlete who kicked near the referees obviously were disappointed about the bad call.
24	h	The athlete who kicked near the referee obviously were disappointed about the bad call.
25	a	The artist who painted the gardens apparently was isolated from society for a long time.
25	b	The artist who painted the garden apparently was isolated from society for a long time.
25	c	The artist who painted the gardens apparently were isolated from society for a long time.
25	d	The artist who painted the garden apparently were isolated from society for a long time.
25	e	The artist who painted in the gardens apparently was isolated from society for a long time.
25	f	The artist who painted in the garden apparently was isolated from society for a long time.
25	g	The artist who painted in the gardens apparently were isolated from society for a long time.
25	h	The artist who painted in the garden apparently were isolated from society for a long time.
26	a	The teacher who called the students obviously was upset about the low grades.
26	b	The teacher who called the student obviously was upset about the low grades.
26	c	The teacher who called the students obviously were upset about the low grades.
26	d	The teacher who called the student obviously were upset about the low grades.
26	e	The teacher who called for the students obviously was upset about the low grades.
26	f	The teacher who called for the student obviously was upset about the low grades.
26	g	The teacher who called for the students obviously were upset about the low grades.
26	h	The teacher who called for the student obviously were upset about the low grades.
27	a	The chef who cooked the onions obviously was growing quite upset about the smell.

27	b	The chef who cooked the onion obviously was growing quite upset about the smell.
27	c	The chef who cooked the onions obviously were growing quite upset about the smell.
27	d	The chef who cooked the onion obviously were growing quite upset about the smell.
27	e	The chef who cooked with the onions obviously was growing quite upset about the smell.
27	f	The chef who cooked with the onion obviously was growing quite upset about the smell.
27	g	The chef who cooked with the onions obviously were growing quite upset about the smell.
27	h	The chef who cooked with the onion obviously were growing quite upset about the smell.
28	a	The host who sat the guests certainly was impressed with all of the colorful decorations.
28	b	The host who sat the guest certainly was impressed with all of the colorful decorations.
28	c	The host who sat the guests certainly were impressed with all of the colorful decorations.
28	d	The host who sat the guest certainly were impressed with all of the colorful decorations.
28	e	The host who sat near the guests certainly was impressed with all of the colorful decorations.
28	f	The host who sat near the guest certainly was impressed with all of the colorful decorations.
28	g	The host who sat near the guests certainly were impressed with all of the colorful decorations.
28	h	The host who sat near the guest certainly were impressed with all of the colorful decorations.
29	a	The stewardess who sat the passengers certainly was very pleased with the long flight.
29	b	The stewardess who sat the passenger certainly was very pleased with the long flight.
29	c	The stewardess who sat the passengers certainly were very pleased with the long flight.
29	d	The stewardess who sat the passenger certainly were very pleased with the long flight.
29	e	The stewardess who sat behind the passengers certainly was very pleased with the long flight.
29	f	The stewardess who sat behind the passenger certainly was very pleased with the long flight.
29	g	The stewardess who sat behind the passengers certainly were very pleased with the long flight.
29	h	The stewardess who sat behind the passenger certainly were very pleased with the long flight.
30	a	The driver who crossed the bridges luckily was very eager to return home.
30	b	The driver who crossed the bridge luckily was very eager to return home.
30	c	The driver who crossed the bridges luckily were very eager to return home.
30	d	The driver who crossed the bridge luckily were very eager to return home.
30	e	The driver who crossed over the bridges luckily was very eager to return home.
30	f	The driver who crossed over the bridge luckily was very eager to return home.
30	g	The driver who crossed over the bridges luckily were very eager to return home.
30	h	The driver who crossed over the bridge luckily were very eager to return home.
31	a	The professor who wrote the tests certainly was mistaken about the final question.
31	b	The professor who wrote the test certainly was mistaken about the final question.
31	c	The professor who wrote the tests certainly were mistaken about the final question.
31	d	The professor who wrote the test certainly were mistaken about the final question.
31	e	The professor who wrote on the tests certainly was mistaken about the final question.

31	f	The professor who wrote on the test certainly was mistaken about the final question.
31	g	The professor who wrote on the tests certainly were mistaken about the final question.
31	h	The professor who wrote on the test certainly were mistaken about the final question.
32	a	The journalist who wrote the articles obviously was unhappy about all the criticism.
32	b	The journalist who wrote the article obviously was unhappy about all the criticism.
32	c	The journalist who wrote the articles obviously were unhappy about all the criticism.
32	d	The journalist who wrote the article obviously were unhappy about all the criticism.
32	e	The journalist who wrote about the articles obviously was unhappy about all the criticism.
32	f	The journalist who wrote about the article obviously was unhappy about all the criticism.
32	g	The journalist who wrote about the articles obviously were unhappy about all the criticism.
32	h	The journalist who wrote about the article obviously were unhappy about all the criticism.
33	a	The robber who broke the safes apparently was hiding from the police on the other side of town.
33	b	The robber who broke the safe apparently was hiding from the police on the other side of town.
33	c	The robber who broke the safes apparently were hiding from the police on the other side of town.
33	d	The robber who broke the safe apparently were hiding from the police on the other side of town.
33	e	The robber who broke into the safes apparently was hiding from the police on the other side of town.
33	f	The robber who broke into the safe apparently was hiding from the police on the other side of town.
33	g	The robber who broke into the safes apparently were hiding from the police on the other side of town.
33	h	The robber who broke into the safe apparently were hiding from the police on the other side of town.
34	a	The dog that chewed the bones probably was pretty lonely the other night.
34	b	The dog that chewed the bone probably was pretty lonely the other night.
34	c	The dog that chewed the bones probably were pretty lonely the other night.
34	d	The dog that chewed the bone probably were pretty lonely the other night.
34	e	The dog that chewed on the bones probably was pretty lonely the other night.
34	f	The dog that chewed on the bone probably was pretty lonely the other night.
34	g	The dog that chewed on the bones probably were pretty lonely the other night.
34	h	The dog that chewed on the bone probably were pretty lonely the other night.
35	a	The coach who trained the athletes definitely was prepared for the championship game.
35	b	The coach who trained the athlete definitely was prepared for the championship game.
35	c	The coach who trained the athletes definitely were prepared for the championship game.
35	d	The coach who trained the athlete definitely were prepared for the championship game.
35	e	The coach who trained with the athletes definitely was prepared for the championship game.
35	f	The coach who trained with the athlete definitely was prepared for the championship game.
35	g	The coach who trained with the athletes definitely were prepared for the championship game.
35	h	The coach who trained with the athlete definitely were prepared for the championship game.
36	a	The child who walked the puppies clearly was happy to be outside in the sunny weather.

36	b	The child who walked the puppy clearly was happy to be outside in the sunny weather.
36	c	The child who walked the puppies clearly were happy to be outside in the sunny weather.
36	d	The child who walked the puppy clearly were happy to be outside in the sunny weather.
36	e	The child who walked by the puppies clearly was happy to be outside in the sunny weather.
36	f	The child who walked by the puppy clearly was happy to be outside in the sunny weather.
36	g	The child who walked by the puppies clearly were happy to be outside in the sunny weather.
36	h	The child who walked by the puppy clearly were happy to be outside in the sunny weather.
37	a	The nurse who called the doctors clearly was nervous about the stressful situation.
37	b	The nurse who called the doctor clearly was nervous about the stressful situation.
37	c	The nurse who called the doctors clearly were nervous about the stressful situation.
37	d	The nurse who called the doctor clearly were nervous about the stressful situation.
37	e	The nurse who called for the doctors clearly was nervous about the stressful situation.
37	f	The nurse who called for the doctor clearly was nervous about the stressful situation.
37	g	The nurse who called for the doctors clearly were nervous about the stressful situation.
37	h	The nurse who called for the doctor clearly were nervous about the stressful situation.
38	a	The boy who walked the dogs suddenly was scared about the loud noises coming from a nearby building.
38	b	The boy who walked the dog suddenly was scared about the loud noises coming from a nearby building.
38	c	The boy who walked the dogs suddenly were scared about the loud noises coming from a nearby building.
38	d	The boy who walked the dog suddenly were scared about the loud noises coming from a nearby building.
38	e	The boy who walked by the dogs suddenly was scared about the loud noises coming from a nearby building.
38	f	The boy who walked by the dog suddenly was scared about the loud noises coming from a nearby building.
38	g	The boy who walked by the dogs suddenly were scared about the loud noises coming from a nearby building.
38	h	The boy who walked by the dog suddenly were scared about the loud noises coming from a nearby building.
39	a	The athlete who ran the races unsurprisingly was upset about losing the championship.
39	b	The athlete who ran the race unsurprisingly was upset about losing the championship.
39	c	The athlete who ran the races unsurprisingly were upset about losing the championship.
39	d	The athlete who ran the race unsurprisingly were upset about losing the championship.
39	e	The athlete who ran in the races unsurprisingly was upset about losing the championship.
39	f	The athlete who ran in the race unsurprisingly was upset about losing the championship.
39	g	The athlete who ran in the races unsurprisingly were upset about losing the championship.
39	h	The athlete who ran in the race unsurprisingly were upset about losing the championship.
40	a	The figher_pilot who flew the planes definitely was scared about the approaching storm.
40	b	The figher_pilot who flew the plane definitely was scared about the approaching storm.
40	c	The figher_pilot who flew the planes definitely were scared about the approaching storm.
40	d	The figher_pilot who flew the plane definitely were scared about the approaching storm.
40	e	The figher_pilot who flew towards the planes definitely was scared about the approaching storm.

40	f	The figher_pilot who flew towards the plane definitely was scared about the approaching storm.
40	g	The figher_pilot who flew towards the planes definitely were scared about the approaching storm.
40	h	The figher_pilot who flew towards the plane definitely were scared about the approaching storm.
41	a	The child who drew the pictures apparently was rather talented at drawing complex shapes.
41	b	The child who drew the picture apparently was rather talented at drawing complex shapes.
41	c	The child who drew the pictures apparently were rather talented at drawing complex shapes.
41	d	The child who drew the picture apparently were rather talented at drawing complex shapes.
41	e	The child who drew on the pictures apparently was rather talented at drawing complex shapes.
41	f	The child who drew on the picture apparently was rather talented at drawing complex shapes.
41	g	The child who drew on the pictures apparently were rather talented at drawing complex shapes.
41	h	The child who drew on the picture apparently were rather talented at drawing complex shapes.
42	a	The researcher who wrote the articles clearly was quite happy with the reviews.
42	b	The researcher who wrote the article clearly was quite happy with the reviews.
42	c	The researcher who wrote the articles clearly were quite happy with the reviews.
42	d	The researcher who wrote the article clearly were quite happy with the reviews.
42	e	The researcher who wrote about the articles clearly was quite happy with the reviews.
42	f	The researcher who wrote about the article clearly was quite happy with the reviews.
42	g	The researcher who wrote about the articles clearly were quite happy with the reviews.
42	h	The researcher who wrote about the article clearly were quite happy with the reviews.
43	a	The child who climbed the trees naturally was upset about breaking his arm after falling.
43	b	The child who climbed the tree naturally was upset about breaking his arm after falling.
43	c	The child who climbed the trees naturally were upset about breaking his arm after falling.
43	d	The child who climbed the tree naturally were upset about breaking his arm after falling.
43	e	The child who climbed on the trees naturally was upset about breaking his arm after falling.
43	f	The child who climbed on the tree naturally was upset about breaking his arm after falling.
43	g	The child who climbed on the trees naturally were upset about breaking his arm after falling.
43	h	The child who climbed on the tree naturally were upset about breaking his arm after falling.
44	a	The actor who read the scripts apparently was interested in auditioning for the lead role.
44	b	The actor who read the script apparently was interested in auditioning for the lead role.
44	c	The actor who read the scripts apparently were interested in auditioning for the lead role.
44	d	The actor who read the script apparently were interested in auditioning for the lead role.
44	e	The actor who read about the scripts apparently was interested in auditioning for the lead role.
44	f	The actor who read about the script apparently was interested in auditioning for the lead role.
44	g	The actor who read about the scripts apparently were interested in auditioning for the lead role.
44	h	The actor who read about the script apparently were interested in auditioning for the lead role.
45	a	The dancer who learned the moves clearly was the best on the stage.

45	b	The dancer who learned the move clearly was the best on the stage.
45	c	The dancer who learned the moves clearly were the best on the stage.
45	d	The dancer who learned the move clearly were the best on the stage.
45	e	The dancer who learned about the moves clearly was the best on the stage.
45	f	The dancer who learned about the move clearly was the best on the stage.
45	g	The dancer who learned about the moves clearly were the best on the stage.
45	h	The dancer who learned about the move clearly were the best on the stage.
46	a	The reporter who met the candidates definitely was nervous about the upcoming debate.
46	b	The reporter who met the candidate definitely was nervous about the upcoming debate.
46	c	The reporter who met the candidates definitely were nervous about the upcoming debate.
46	d	The reporter who met the candidate definitely were nervous about the upcoming debate.
46	e	The reporter who met with the candidates definitely was nervous about the upcoming debate.
46	f	The reporter who met with the candidate definitely was nervous about the upcoming debate.
46	g	The reporter who met with the candidates definitely were nervous about the upcoming debate.
46	h	The reporter who met with the candidate definitely were nervous about the upcoming debate.
47	a	The fan who met the musicians certainly was displeased with the horrible performance.
47	b	The fan who met the musician certainly was displeased with the horrible performance.
47	c	The fan who met the musicians certainly were displeased with the horrible performance.
47	d	The fan who met the musician certainly were displeased with the horrible performance.
47	e	The fan who met with the musicians certainly was displeased with the horrible performance.
47	f	The fan who met with the musician certainly was displeased with the horrible performance.
47	g	The fan who met with the musicians certainly were displeased with the horrible performance.
47	h	The fan who met with the musician certainly were displeased with the horrible performance.
48	a	The journalist who met the diplomats undoubtedly was confused about the current political situation.
48	b	The journalist who met the diplomat undoubtedly was confused about the current political situation.
48	c	The journalist who met the diplomats undoubtedly were confused about the current political situation.
48	d	The journalist who met the diplomat undoubtedly were confused about the current political situation.
48	e	The journalist who met with the diplomats undoubtedly was confused about the current political situation.
48	f	The journalist who met with the diplomat undoubtedly was confused about the current political situation.
48	g	The journalist who met with the diplomats undoubtedly were confused about the current political situation.
48	h	The journalist who met with the diplomat undoubtedly were confused about the current political situation.

1.2 Experiment 2 Materials

1	a	The manager who the employees greeted evidently was unhappy about all the noise.
1	b	The manager who the employee greeted evidently was unhappy about all the noise.

1	c	The manager who the employees greeted evidently were unhappy about all the noise.
1	d	The manager who the employee greeted evidently were unhappy about all the noise.
1	e	The manager who greeted the employees evidently was unhappy about all the noise.
1	f	The manager who greeted the employee evidently was unhappy about all the noise.
1	g	The manager who greeted the employees evidently were unhappy about all the noise.
1	h	The manager who greeted the employee unsurprisingly were unhappy about all the noise.
2	a	The boy who the girls mocked probably was expelled from school for bad behavior.
2	b	The boy who the girl mocked probably was expelled from school for bad behavior.
2	c	The boy who the girls mocked probably were expelled from school for bad behavior.
2	d	The boy who the girl mocked probably were expelled from school for bad behavior.
2	e	The boy who mocked the girls probably was expelled from school for bad behavior.
2	f	The boy who mocked the girl probably was expelled from school for bad behavior.
2	g	The boy who mocked the girls probably were expelled from school for bad behavior.
2	h	The boy who mocked the girl probably were expelled from school for bad behavior.
3	a	The teacher who the teenagers annoyed apparently was dissatisfied with the low-paying job.
3	b	The teacher who the teenager annoyed apparently was dissatisfied with the low-paying job.
3	c	The teacher who the teenagers annoyed apparently were dissatisfied with the low-paying job.
3	d	The teacher who the teenager annoyed apparently were dissatisfied with the low-paying job.
3	e	The teacher who annoyed the teenagers apparently was dissatisfied with the low-paying job.
3	f	The teacher who annoyed the teenager apparently was dissatisfied with the low-paying job.
3	g	The teacher who annoyed the teenagers apparently were dissatisfied with the low-paying job.
3	h	The teacher who annoyed the teenager apparently were dissatisfied with the low-paying job.
4	a	The policeman who the criminals punched evidently was suspended without pay for not following the rules.
4	b	The policeman who the criminal punched evidently was suspended without pay for not following the rules.
4	c	The policeman who the criminals punched evidently were suspended without pay for not following the rules.
4	d	The policeman who the criminal punched evidently were suspended without pay for not following the rules.
4	e	The policeman who punched the criminals evidently was suspended without pay for not following the rules.
4	f	The policeman who punched the criminal evidently was suspended without pay for not following the rules.
4	g	The policeman who punched the criminals evidently were suspended without pay for not following the rules.
4	h	The policeman who punched the criminal evidently were suspended without pay for not following the rules.
5	a	The scientist who the technicians notified obviously was worried about the smell of gas.
5	b	The scientist who the technician notified obviously was worried about the smell of gas.
5	c	The scientist who the technicians notified obviously were worried about the smell of gas.
5	d	The scientist who the technician notified obviously were worried about the smell of gas.
5	e	The scientist who notified the technicians obviously was worried about the smell of gas.
5	f	The scientist who notified the technician obviously was worried about the smell of gas.

5	g	The scientist who notified the technicians obviously were worried about the smell of gas.
5	h	The scientist who notified the technician obviously were worried about the smell of gas.
6	a	The senator who the protestors emailed apparently was investigated for tax fraud last year.
6	b	The senator who the protestor emailed apparently was investigated for tax fraud last year.
6	c	The senator who the protestors emailed apparently were investigated for tax fraud last year.
6	d	The senator who the protestor emailed apparently were investigated for tax fraud last year.
6	e	The senator who emailed the protestors apparently was investigated for tax fraud last year.
6	f	The senator who emailed the protestor apparently was investigated for tax fraud last year.
6	g	The senator who emailed the protestors apparently were investigated for tax fraud last year.
6	h	The senator who emailed the protestor apparently were investigated for tax fraud last year.
7	a	The celebrity who the photographers sued likely was unaware of the archaic laws in California.
7	b	The celebrity who the photographer sued likely was unaware of the archaic laws in California.
7	c	The celebrity who the photographers sued likely were unaware of the archaic laws in California.
7	d	The celebrity who the photographer sued likely were unaware of the archaic laws in California.
7	e	The celebrity who sued the photographers likely was unaware of the archaic laws in California.
7	f	The celebrity who sued the photographer likely was unaware of the archaic laws in California.
7	g	The celebrity who sued the photographers likely were unaware of the archaic laws in California.
7	h	The celebrity who sued the photographer likely were unaware of the archaic laws in California.
8	a	The doctor who the nurses consulted unsurprisingly was worried about the bad news.
8	b	The doctor who the nurse consulted unsurprisingly was worried about the bad news.
8	c	The doctor who the nurses consulted unsurprisingly were worried about the bad news.
8	d	The doctor who the nurse consulted unsurprisingly were worried about the bad news.
8	e	The doctor who consulted the nurses unsurprisingly was worried about the bad news.
8	f	The doctor who consulted the nurse unsurprisingly was worried about the bad news.
8	g	The doctor who consulted the nurses unsurprisingly were worried about the bad news.
8	h	The doctor who consulted the nurse unsurprisingly were worried about the bad news.
9	a	The soldier who the diplomats accompanied suddenly was surrounded by a team of insurgents.
9	b	The soldier who the diplomat accompanied suddenly was surrounded by a team of insurgents.
9	c	The soldier who the diplomats accompanied suddenly were surrounded by a team of insurgents.
9	d	The soldier who the diplomat accompanied suddenly were surrounded by a team of insurgents.
9	e	The soldier who accompanied the diplomats suddenly was surrounded by a team of insurgents.
9	f	The soldier who accompanied the diplomat suddenly was surrounded by a team of insurgents.
9	g	The soldier who accompanied the diplomats suddenly were surrounded by a team of insurgents.
9	h	The soldier who accompanied the diplomat suddenly were surrounded by a team of insurgents.
10	a	The waitress who the guests offended certainly was surprised by the large tip.
10	b	The waitress who the guest offended certainly was surprised by the large tip.

10	c	The waitress who the guests offended certainly were surprised by the large tip.
10	d	The waitress who the guest offended certainly were surprised by the large tip.
10	e	The waitress who offended the guests certainly was surprised by the large tip.
10	f	The waitress who offended the guest certainly was surprised by the large tip.
10	g	The waitress who offended the guests certainly were surprised by the large tip.
10	h	The waitress who offended the guest certainly were surprised by the large tip.
11	a	The student who the teachers nominated obviously was excited for the start of summer.
11	b	The student who the teacher nominated obviously was excited for the start of summer.
11	c	The student who the teachers nominated obviously were excited for the start of summer.
11	d	The student who the teacher nominated obviously were excited for the start of summer.
11	e	The student who nominated the teachers obviously was excited for the start of summer.
11	f	The student who nominated the teacher obviously was excited for the start of summer.
11	g	The student who nominated the teachers obviously were excited for the start of summer.
11	h	The student who nominated the teacher obviously were excited for the start of summer.
12	a	The bully who the boys antagonized allegedly was accused of harassing a group of girls.
12	b	The bully who the boy antagonized allegedly was accused of harassing a group of girls.
12	c	The bully who the boys antagonized allegedly were accused of harassing a group of girls.
12	d	The bully who the boy antagonized allegedly were accused of harassing a group of girls.
12	e	The bully who antagonized the boys allegedly was accused of harassing a group of girls.
12	f	The bully who antagonized the boy allegedly was accused of harassing a group of girls.
12	g	The bully who antagonized the boys allegedly were accused of harassing a group of girls.
12	h	The bully who antagonized the boy allegedly were accused of harassing a group of girls.
13	a	The technician who the workers alerted apparently was unaware about the noise in the basement.
13	b	The technician who the worker alerted apparently was worried about the noise in the basement.
13	c	The technician who the workers alerted apparently were worried about the noise in the basement.
13	d	The technician who the worker alerted apparently were worried about the noise in the basement.
13	e	The technician who alerted the workers apparently was worried about the noise in the basement.
13	f	The technician who alerted the worker apparently was worried about the noise in the basement.
13	g	The technician who alerted the workers apparently were worried about the noise in the basement.
13	h	The technician who alerted the worker apparently were worried about the noise in the basement.
14	a	The aunt who the boys hugged definitely was tired from the long trip across the state.
14	b	The aunt who the boy hugged definitely was tired from the long trip across the state.
14	c	The aunt who the boys hugged definitely were tired from the long trip across the state.
14	d	The aunt who the boy hugged definitely were tired from the long trip across the state.
14	e	The aunt who hugged the boys definitely was tired from the long trip across the state.
14	f	The aunt who hugged the boy definitely was tired from the long trip across the state.

14	g	The aunt who hugged the boys definitely were tired from the long trip across the state.
14	h	The aunt who hugged the boy definitely were tired from the long trip across the state.
15	a	The professor who the students inspired certainly was happy about how the final projects turned out.
15	b	The professor who the student inspired certainly was happy about how the final projects turned out.
15	c	The professor who the students inspired certainly were happy about how the final projects turned out.
15	d	The professor who the student inspired certainly were happy about how the final projects turned out.
15	e	The professor who inspired the students certainly was happy about how the final projects turned out.
15	f	The professor who inspired the student certainly was happy about how the final projects turned out.
15	g	The professor who inspired the students certainly were happy about how the final projects turned out.
15	h	The professor who inspired the student certainly were happy about how the final projects turned out.
16	a	The secretary who the employees assisted definitely was nervous about the upcoming merger.
16	b	The secretary who the employee assisted definitely was nervous about the upcoming merger.
16	c	The secretary who the employees assisted definitely were nervous about the upcoming merger.
16	d	The secretary who the employee assisted definitely were nervous about the upcoming merger.
16	e	The secretary who assisted the employees definitely was nervous about the upcoming merger.
16	f	The secretary who assisted the employee definitely was nervous about the upcoming merger.
16	g	The secretary who assisted the employees definitely were nervous about the upcoming merger.
16	h	The secretary who assisted the employee definitely were nervous about the upcoming merger.
17	a	The defendant who the witnesses described allegedly was accused of stealing from a restaurant.
17	b	The defendant who the witness described allegedly was accused of stealing from a restaurant.
17	c	The defendant who the witnesses described allegedly were accused of stealing from a restaurant.
17	d	The defendant who the witness described allegedly were accused of stealing from a restaurant.
17	e	The defendant who described the witnesses allegedly was accused of stealing from a restaurant.
17	f	The defendant who described the witness allegedly was accused of stealing from a restaurant.
17	g	The defendant who described the witnesses allegedly were accused of stealing from a restaurant.
17	h	The defendant who described the witness allegedly were accused of stealing from a restaurant.
18	a	The celebrity who the journalists insulted certainly was upset about the recent accusations.
18	b	The celebrity who the journalist insulted certainly was upset about the recent accusations.
18	c	The celebrity who the journalists insulted certainly were upset about the recent accusations.
18	d	The celebrity who the journalist insulted certainly were upset about the recent accusations.
18	e	The celebrity who insulted the journalists certainly was upset about the recent accusations.
18	f	The celebrity who insulted the journalist certainly was upset about the recent accusations.
18	g	The celebrity who insulted the journalists certainly were upset about the recent accusations.
18	h	The celebrity who insulted the journalist certainly were upset about the recent accusations.
19	a	The policeman who the protestors provoked apparently was upset about the new laws.
19	b	The policeman who the protestor provoked apparently was upset about the new laws.

19	c	The policeman who the protestors provoked apparently were upset about the new laws.
19	d	The policeman who the protestor provoked apparently were upset about the new laws.
19	e	The policeman who provoked the protestors apparently was upset about the new laws.
19	f	The policeman who provoked the protestor apparently was upset about the new laws.
19	g	The policeman who provoked the protestors apparently were upset about the new laws.
19	h	The policeman who provoked the protestor apparently were upset about the new laws.
20	a	The manager who the interns humiliated probably was unaware that the joke was offensive.
20	b	The manager who the intern humiliated probably was unaware that the joke was offensive.
20	c	The manager who the interns humiliated probably were unaware that the joke was offensive.
20	d	The manager who the intern humiliated probably were unaware that the joke was offensive.
20	e	The manager who humiliated the interns probably was unaware that the joke was offensive.
20	f	The manager who humiliated the intern probably was unaware that the joke was offensive.
20	g	The manager who humiliated the interns probably were unaware that the joke was offensive.
20	h	The manager who humiliated the intern probably were unaware that the joke was offensive.
21	a	The engineer who the architects recommended recently was promoted to a new regional office.
21	b	The engineer who the architect recommended recently was promoted to a new regional office.
21	c	The engineer who the architects recommended recently were promoted to a new regional office.
21	d	The engineer who the architect recommended recently were promoted to a new regional office.
21	e	The engineer who recommended the architects recently was promoted to a new regional office.
21	f	The engineer who recommended the architect recently was promoted to a new regional office.
21	g	The engineer who recommended the architects recently were promoted to a new regional office.
21	h	The engineer who recommended the architect recently were promoted to a new regional office.
22	a	The manager who the secretaries impressed probably was nervous about the new job.
22	b	The manager who the secretary encouraged probably was nervous about the new job.
22	c	The manager who the secretaries encouraged probably were nervous about the new job.
22	d	The manager who the secretary encouraged probably were nervous about the new job.
22	e	The manager who impressed the secretaries probably was nervous about the new job.
22	f	The manager who impressed the secretary probably was nervous about the new job.
22	g	The manager who impressed the secretaries probably were nervous about the new job.
22	h	The manager who impressed the secretary probably were nervous about the new job.
23	a	The actor who the journalists loathed apparently was cast as the lead in a new thriller.
23	b	The actor who the journalist loathed apparently was cast as the lead in a new thriller.
23	c	The actor who the journalists loathed apparently were cast as the lead in a new thriller.
23	d	The actor who the journalist loathed apparently were cast as the lead in a new thriller.
23	e	The actor who loathed the journalists apparently was cast as the lead in a new thriller.
23	f	The actor who loathed the journalist apparently was cast as the lead in a new thriller.

23	g	The actor who loathed the journalists apparently were cast as the lead in a new thriller.
23	h	The actor who loathed the journalist apparently were cast as the lead in a new thriller.
24	a	The athlete who the reporters disliked evidently was banned from the upcoming Olympic games.
24	b	The athlete who the reporter disliked evidently was banned from the upcoming Olympic games.
24	c	The athlete who the reporters disliked evidently were banned from the upcoming Olympic games.
24	d	The athlete who the reporter disliked evidently were banned from the upcoming Olympic games.
24	e	The athlete who disliked the reporters evidently was banned from the upcoming Olympic games.
24	f	The athlete who disliked the reporter evidently was banned from the upcoming Olympic games.
24	g	The athlete who disliked the reporters evidently were banned from the upcoming Olympic games.
24	h	The athlete who disliked the reporter evidently were banned from the upcoming Olympic games.
25	a	The boy who the girls hated apparently was afraid of what they might think of him.
25	b	The boy who the girl hated apparently was afraid of what they might think of him.
25	c	The boy who the girls hated apparently were afraid of what they might think of him.
25	d	The boy who the girl hated apparently were afraid of what they might think of him.
25	e	The boy who hated the girls apparently was afraid of what they might think of him.
25	f	The boy who hated the girl apparently was afraid of what they might think of him.
25	g	The boy who hated the girls apparently were afraid of what they might think of him.
25	h	The boy who hated the girl apparently were afraid of what they might think of him.
26	a	The architect who the engineers praised apparently was offered a job at another company.
26	b	The architect who the engineer praised apparently was offered a job at another company.
26	c	The architect who the engineers praised apparently were offered a job at another company.
26	d	The architect who the engineer praised apparently were offered a job at another company.
26	e	The architect who praised the engineers apparently was offered a job at another company.
26	f	The architect who praised the engineer apparently was offered a job at another company.
26	g	The architect who praised the engineers apparently were offered a job at another company.
26	h	The architect who praised the engineer apparently were offered a job at another company.
27	a	The congressman who the senators endorsed allegedly was blackmailed with some incriminating photos.
27	b	The congressman who the senator endorsed allegedly was blackmailed with some incriminating photos.
27	c	The congressman who the senators endorsed allegedly were blackmailed with some incriminating photos.
27	d	The congressman who the senator endorsed allegedly were blackmailed with some incriminating photos.
27	e	The congressman who endorsed the senators allegedly was blackmailed with some incriminating photos.
27	f	The congressman who endorsed the senator allegedly was blackmailed with some incriminating photos.
27	g	The congressman who endorsed the senators allegedly were blackmailed with some incriminating photos.
27	h	The congressman who endorsed the senator allegedly were blackmailed with some incriminating photos.
28	a	The businessman who the journalists blackmailed allegedly was caught having an affair with another woman.
28	b	The businessman who the journalist blackmailed allegedly was caught having an affair with another woman.

28	c	The businessman who the journalists blackmailed allegedly were caught having an affair with another woman.
28	d	The businessman who the journalist blackmailed allegedly were caught having an affair with another woman.
28	e	The businessman who blackmailed the journalists allegedly was caught having an affair with another woman.
28	f	The businessman who blackmailed the journalist allegedly was caught having an affair with another woman.
28	g	The businessman who blackmailed the journalists allegedly were caught having an affair with another woman.
28	h	The businessman who blackmailed the journalist allegedly were caught having an affair with another woman.
29	a	The politician who the reporters avoided reportedly was hypocritical in condemning the lobbyists.
29	b	The politician who the reporter avoided reportedly was hypocritical in condemning the lobbyists.
29	c	The politician who the reporters avoided reportedly were hypocritical in condemning the lobbyists.
29	d	The politician who the reporter avoided reportedly were hypocritical in condemning the lobbyists.
29	e	The politician who avoided the reporters reportedly was hypocritical in condemning the lobbyists.
29	f	The politician who avoided the reporter reportedly was hypocritical in condemning the lobbyists.
29	g	The politician who avoided the reporters reportedly were hypocritical in condemning the lobbyists.
29	h	The politician who avoided the reporter reportedly were hypocritical in condemning the lobbyists.
30	a	The student who the teachers helped evidently was suspended after failing several classes.
30	b	The student who the teacher helped evidently was suspended after failing several classes.
30	c	The student who the teachers helped evidently were suspended after failing several classes.
30	d	The student who the teacher helped evidently were suspended after failing several classes.
30	e	The student who helped the teachers evidently was suspended after failing several classes.
30	f	The student who helped the teacher evidently was suspended after failing several classes.
30	g	The student who helped the teachers evidently were suspended after failing several classes.
30	h	The student who helped the teacher evidently were suspended after failing several classes.
31	a	The sergeant who the soldiers contacted typically was calm during very stressful moments.
31	b	The sergeant who the solider contacted typically was calm during very stressful moments.
31	c	The sergeant who the soldiers contacted typically were calm during very stressful moments.
31	d	The sergeant who the solider contacted typically were calm during very stressful moments.
31	e	The sergeant who contacted the soldiers typically was calm during very stressful moments.
31	f	The sergeant who contacted the solider typically was calm during very stressful moments.
31	g	The sergeant who contacted the soldiers typically were calm during very stressful moments.
31	h	The sergeant who contacted the solider typically were calm during very stressful moments.
32	a	The CEO who the accountants advised probably was happy with the recent corporate merger.
32	b	The CEO who the accountant advised probably was happy with the recent corporate merger.
32	c	The CEO who the accountants advised probably were happy with the recent corporate merger.
32	d	The CEO who the accountant advised probably were happy with the recent corporate merger.
32	e	The CEO who advised the accountants probably was happy with the recent corporate merger.
32	f	The CEO who advised the accountant probably was happy with the recent corporate merger.

32	g	The CEO who advised the accountants probably were happy with the recent corporate merger.
32	h	The CEO who advised the accountant probably were happy with the recent corporate merger.
33	a	The reporter who the photographers followed obviously was prepared for the treacherous journey.
33	b	The reporter who the photographer followed obviously was prepared for the treacherous journey.
33	c	The reporter who the photographers followed obviously were prepared for the treacherous journey.
33	d	The reporter who the photographer followed obviously were prepared for the treacherous journey.
33	e	The reporter who followed the photographers obviously was prepared for the treacherous journey.
33	f	The reporter who followed the photographer obviously was prepared for the treacherous journey.
33	g	The reporter who followed the photographers obviously were prepared for the treacherous journey.
33	h	The reporter who followed the photographer obviously were prepared for the treacherous journey.
34	a	The candidate who the reporters chastised probably was angry about the false accusations.
34	b	The candidate who the reporter chastised probably was angry about the false accusations.
34	c	The candidate who the reporters chastised probably were angry about the false accusations.
34	d	The candidate who the reporter chastised probably were angry about the false accusations.
34	e	The candidate who chastised the reporters probably was angry about the false accusations.
34	f	The candidate who chastised the reporter probably was angry about the false accusations.
34	g	The candidate who chastised the reporters probably were angry about the false accusations.
34	h	The candidate who chastised the reporter probably were angry about the false accusations.
35	a	The secretary who the interns assisted obviously was excited about all of the new opportunities.
35	b	The secretary who the intern assisted obviously was excited about all of the new opportunities.
35	c	The secretary who the interns assisted obviously were excited about all of the new opportunities.
35	d	The secretary who the intern assisted obviously were excited about all of the new opportunities.
35	e	The secretary who the interns assisted obviously was excited about all of the new opportunities.
35	f	The secretary who assisted the intern obviously was excited about all of the new opportunities.
35	g	The secretary who assisted the interns obviously were excited about all of the new opportunities.
35	h	The secretary who assisted the intern obviously were excited about all of the new opportunities.
36	a	The ambassador who the soldiers accompanied obviously was skeptical about the recent allegations.
36	b	The ambassador who the soldier accompanied obviously was skeptical about the recent allegations.
36	c	The ambassador who the soldiers accompanied obviously were skeptical about the recent allegations.
36	d	The ambassador who the soldier accompanied obviously were skeptical about the recent allegations.
36	e	The ambassador who accompanied the soldiers obviously was skeptical about the recent allegations.
36	f	The ambassador who accompanied the soldier obviously was skeptical about the recent allegations.
36	g	The ambassador who accompanied the soldiers obviously were skeptical about the recent allegations.
36	h	The ambassador who accompanied the soldier obviously were skeptical about the recent allegations.
37	a	The officer who the detectives aided reportedly was suspicious of the noise coming from the empty building.
37	b	The officer who the detective aided reportedly was suspicious of the noise coming from the empty building.

37	c	The officer who the detectives aided reportedly were suspicious of the noise coming from the empty building.
37	d	The officer who the detective aided reportedly were suspicious of the noise coming from the empty building.
37	e	The officer who aided the detectives reportedly was suspicious of the noise coming from the empty building.
37	f	The officer who aided the detective reportedly was suspicious of the noise coming from the empty building.
37	g	The officer who aided the detectives reportedly were suspicious of the noise coming from the empty building.
37	h	The officer who aided the detective reportedly were suspicious of the noise coming from the empty building.
38	a	The sergeant who the corporals advised clearly was unhappy with the training regimen.
38	b	The sergeant who the corporal advised clearly was unhappy with the training regimen.
38	c	The sergeant who the corporals advised clearly were unhappy with the training regimen.
38	d	The sergeant who the corporal advised clearly were unhappy with the training regimen.
38	e	The sergeant who advised the corporals clearly was unhappy with the training regimen.
38	f	The sergeant who advised the corporal clearly was unhappy with the training regimen.
38	g	The sergeant who advised the corporals clearly were unhappy with the training regimen.
38	h	The sergeant who advised the corporal clearly were unhappy with the training regimen.
39	a	The coach who the athletes nominated probably was surprised by the good news.
39	b	The coach who the athlete nominated probably was surprised by the good news.
39	c	The coach who the athletes nominated probably were surprised by the good news.
39	d	The coach who the athlete nominated probably were surprised by the good news.
39	e	The coach who nominated the athletes probably was surprised by the good news.
39	f	The coach who nominated the athlete probably was surprised by the good news.
39	g	The coach who nominated the athletes probably were surprised by the good news.
39	h	The coach who nominated the athlete probably were surprised by the good news.
40	a	The student who the professors recommended clearly was knowledgeable about current foreign affairs.
40	b	The student who the professor recommended clearly was knowledgeable about current foreign affairs.
40	c	The student who the professors recommended clearly were knowledgeable about current foreign affairs.
40	d	The student who the professor recommended clearly were knowledgeable about current foreign affairs.
40	e	The student who recommended the professors clearly was knowledgeable about current foreign affairs.
40	f	The student who recommended the professor clearly was knowledgeable about current foreign affairs.
40	g	The student who recommended the professors clearly were knowledgeable about current foreign affairs.
40	h	The student who recommended the professor clearly were knowledgeable about current foreign affairs.
41	a	The assistant who the secretaries disliked apparently was annoyed by all of the gossip in the office.
41	b	The assistant who the secretary disliked apparently was annoyed by all of the gossip in the office.
41	c	The assistant who the secretaries disliked apparently were annoyed by all of the gossip in the office.
41	d	The assistant who the secretary disliked apparently were annoyed by all of the gossip in the office.
41	e	The assistant who disliked the secretaries apparently was annoyed by all of the gossip in the office.
41	f	The assistant who disliked the secretary apparently was annoyed by all of the gossip in the office.

41	g	The assistant who disliked the secretaries apparently were annoyed by all of the gossip in the office.
41	h	The assistant who disliked the secretary apparently were annoyed by all of the gossip in the office.
42	a	The woman who the housewives insulted apparently was amused by the reaction from the guests.
42	b	The woman who the housewife insulted apparently was amused by the reaction from the guests.
42	c	The woman who the housewives insulted apparently were amused by the reaction from the guests.
42	d	The woman who the housewife insulted apparently were amused by the reaction from the guests.
42	e	The woman who insulted the housewives apparently was amused by the reaction from the guests.
42	f	The woman who insulted the housewife apparently was amused by the reaction from the guests.
42	g	The woman who insulted the housewives apparently were amused by the reaction from the guests.
42	h	The woman who insulted the housewife apparently were amused by the reaction from the guests.
43	a	The businessman who the clients annoyed clearly was unhappy with the final product.
43	b	The businessman who the client annoyed clearly was unhappy with the final product.
43	c	The businessman who the clients annoyed clearly were unhappy with the final product.
43	d	The businessman who the client annoyed clearly were unhappy with the final product.
43	e	The businessman who annoyed the clients clearly was unhappy with the final product.
43	f	The businessman who annoyed the client clearly was unhappy with the final product.
43	g	The businessman who annoyed the clients clearly were unhappy with the final product.
43	h	The businessman who annoyed the client clearly were unhappy with the final product.
44	a	The candidate who the reporters humiliated surprisingly was elected into office for a second term.
44	b	The candidate who the reporter humiliated surprisingly was elected into office for a second term.
44	c	The candidate who the reporters humiliated surprisingly were elected into office for a second term.
44	d	The candidate who the reporter humiliated surprisingly were elected into office for a second term.
44	e	The candidate who humiliated the reporters surprisingly was elected into office for a second term.
44	f	The candidate who humiliated the reporter surprisingly was elected into office for a second term.
44	g	The candidate who humiliated the reporters surprisingly were elected into office for a second term.
44	h	The candidate who humiliated the reporter surprisingly were elected into office for a second term.
45	a	The detective who the lawyers notified allegedly was involved in a tax fraud investigation.
45	b	The detective who the lawyer notified allegedly was involved in a tax fraud investigation.
45	c	The detective who the lawyers notified allegedly were involved in a tax fraud investigation.
45	d	The detective who the lawyer notified allegedly were involved in a tax fraud investigation.
45	e	The detective who notified the lawyers allegedly was involved in a tax fraud investigation.
45	f	The detective who notified the lawyer allegedly was involved in a tax fraud investigation.
45	g	The detective who notified the lawyers allegedly were involved in a tax fraud investigation.
45	h	The detective who notified the lawyer allegedly were involved in a tax fraud investigation.
46	a	The manager who the employees emailed definitely was pleased with the positive responses.
46	b	The manager who the employee emailed definitely was pleased with the positive responses.

46	c	The manager who the employees emailed definitely were pleased with the positive responses.
46	d	The manager who the employee emailed definitely were pleased with the positive responses.
46	e	The manager who emailed the employees definitely was pleased with the positive responses.
46	f	The manager who emailed the employee definitely was pleased with the positive responses.
46	g	The manager who emailed the employees definitely were pleased with the positive responses.
46	h	The manager who emailed the employee definitely were pleased with the positive responses.
47	a	The accountant who the lawyers contacted evidently was unwilling to cooperate with the police.
47	b	The accountant who the lawyer contacted evidently was unwilling to cooperate with the police.
47	c	The accountant who the lawyers contacted evidently were unwilling to cooperate with the police.
47	d	The accountant who the lawyer contacted evidently were unwilling to cooperate with the police.
47	e	The accountant who contacted the lawyers evidently was unwilling to cooperate with the police.
47	f	The accountant who contacted the lawyer evidently was unwilling to cooperate with the police.
47	g	The accountant who contacted the lawyers evidently were unwilling to cooperate with the police.
47	h	The accountant who contacted the lawyer evidently were unwilling to cooperate with the police.
48	a	The detective who the officers accompanied apparently was notified about the crime several days earlier.
48	b	The detective who the officer accompanied apparently was notified about the crime several days earlier.
48	c	The detective who the officers accompanied apparently were notified about the crime several days earlier.
48	d	The detective who the officer accompanied apparently were notified about the crime several days earlier.
48	e	The detective who accompanied the officers apparently was notified about the crime several days earlier.
48	f	The detective who accompanied the officer apparently was notified about the crime several days earlier.
48	g	The detective who accompanied the officers apparently were notified about the crime several days earlier.
48	h	The detective who accompanied the officer apparently were notified about the crime several days earlier.

1.3 Experiment 3 Materials

1	a	The house that had been built by the worker sadly was falling into disrepair.
1	b	The house that had been built by the workers sadly was falling into disrepair.
1	c	The house that had been built by the worker sadly were falling into disrepair.
1	d	The house that had been built by the workers sadly were falling into disrepair.
2	a	The statue that had been destroyed by the teenager surprisingly was insured for only a quarter of a million dollars.
2	b	The statue that had been destroyed by the teenagers surprisingly was insured for only a quarter of a million dollars.
2	c	The statue that had been destroyed by the teenager surprisingly were insured for only a quarter of a million dollars.
2	d	The statue that had been destroyed by the teenagers surprisingly were insured for only a quarter of a million dollars.
3	a	The mural that had been painted by the student unfortunately was covered in graffiti by the start of the next semester.
3	b	The mural that had been painted by the students unfortunately was covered in graffiti by the start of the next semester.
3	c	The mural that had been painted by the student unfortunately were covered in graffiti by the start of the next semester.

3	d	The mural that had been painted by the students unfortunately were covered in graffiti by the start of the next semester.
4	a	The report that had been written by the diplomat somehow was lost in transit across the border.
4	b	The report that had been written by the diplomats somehow was lost in transit across the border.
4	c	The report that had been written by the diplomat somehow were lost in transit across the border.
4	d	The report that had been written by the diplomats somehow were lost in transit across the border.
5	a	The room that had been cleaned by the janitor thankfully was cleared of the awful stench.
5	b	The room that had been cleaned by the janitors thankfully was cleared of the awful stench.
5	c	The room that had been cleaned by the janitor thankfully were cleared of the awful stench.
5	d	The room that had been cleaned by the janitors thankfully were cleared of the awful stench.
6	a	The meeting that had been delayed by the employee eventually was rescheduled for later the next day.
6	b	The meeting that had been delayed by the employees eventually was rescheduled for later the next day.
6	c	The meeting that had been delayed by the employee eventually were rescheduled for later the next day.
6	d	The meeting that had been delayed by the employees eventually were rescheduled for later the next day.
7	a	The paragraph that had been rewritten by the author ultimately was removed from the article.
7	b	The paragraph that had been rewritten by the authors ultimately was removed from the article.
7	c	The paragraph that had been rewritten by the author ultimately were removed from the article.
7	d	The paragraph that had been rewritten by the authors ultimately were removed from the article.
8	a	The apartment that had been occupied by the student fortunately was cleaned before it was inspected.
8	b	The apartment that had been occupied by the students fortunately was cleaned before it was inspected.
8	c	The apartment that had been occupied by the student fortunately were cleaned before it was inspected.
8	d	The apartment that had been occupied by the students fortunately were cleaned before it was inspected.
9	a	The toy that had been hidden by the kid quickly was found by impatient babysitter.
9	b	The toy that had been hidden by the kids quickly was found by impatient babysitter.
9	c	The toy that had been hidden by the kid quickly were found by impatient babysitter.
9	d	The toy that had been hidden by the kids quickly were found by impatient babysitter.
10	a	The phenomenon that had been studied by the scientist finally was beginning to be well-understood.
10	b	The phenomenon that had been studied by the scientists finally was beginning to be well-understood.
10	c	The phenomenon that had been studied by the scientist finally were beginning to be well-understood.
10	d	The phenomenon that had been studied by the scientists finally were beginning to be well-understood.
11	a	The car that had been stolen by the criminal fortunately was found in an empty parking lot.
11	b	The car that had been stolen by the criminals fortunately was found in an empty parking lot.
11	c	The car that had been stolen by the criminal fortunately were found in an empty parking lot.
11	d	The car that had been stolen by the criminals fortunately were found in an empty parking lot.
12	a	The building that had been damaged by the terrorist slowly was rebuilt by a dedicated team of volunteers.
12	b	The building that had been damaged by the terrorists slowly was rebuilt by a dedicated team of volunteers.
12	c	The building that had been damaged by the terrorist slowly were rebuilt by a dedicated team of volunteers.

12	d	The building that had been damaged by the terrorists slowly were rebuilt by a dedicated team of volunteers.
13	a	The cave that had been blocked by the boulder still was unexplored by the end of the 20th century.
13	b	The cave that had been blocked by the boulders still was unexplored by the end of the 20th century.
13	c	The cave that had been blocked by the boulder still were unexplored by the end of the 20th century.
13	d	The cave that had been blocked by the boulders still were unexplored by the end of the 20th century.
14	a	The report that had been created by the investigator accidentally was deleted by one of the new hires.
14	b	The report that had been created by the investigators accidentally was deleted by one of the new hires.
14	c	The report that had been created by the investigator accidentally were deleted by one of the new hires.
14	d	The report that had been created by the investigators accidentally were deleted by one of the new hires.
15	a	The meal that had been prepared by the host quickly was devoured before the desert was served.
15	b	The meal that had been prepared by the hosts quickly was devoured before the desert was served.
15	c	The meal that had been prepared by the host quickly were devoured before the desert was served.
15	d	The meal that had been prepared by the hosts quickly were devoured before the desert was served.
16	a	The prototype that had been inspected by the engineer unfortunately was discovered to have a major flaw.
16	b	The prototype that had been inspected by the engineers unfortunately was discovered to have a major flaw.
16	c	The prototype that had been inspected by the engineer unfortunately were discovered to have a major flaw.
16	d	The prototype that had been inspected by the engineers unfortunately were discovered to have a major flaw.
17	a	The blueprint that had been designed by the architect quickly was fixed when a safety hazard was discovered.
17	b	The blueprint that had been designed by the architects quickly was fixed when a safety hazard was discovered.
17	c	The blueprint that had been designed by the architect quickly were fixed when a safety hazard was discovered.
17	d	The blueprint that had been designed by the architects quickly were fixed when a safety hazard was discovered.
18	a	The engine that had been repaired by the mechanic thankfully was running good as new by the next day.
18	b	The engine that had been repaired by the mechanics thankfully was running good as new by the next day.
18	c	The engine that had been repaired by the mechanic thankfully were running good as new by the next day.
18	d	The engine that had been repaired by the mechanics thankfully were running good as new by the next day.
19	a	The skyscraper that had been designed by the architect deservedly was given many awards.
19	b	The skyscraper that had been designed by the architects deservedly was given many awards.
19	c	The skyscraper that had been designed by the architect deservedly were given many awards.
19	d	The skyscraper that had been designed by the architects deservedly were given many awards.
20	a	The report that had been written by the professor surprisingly was not mentioned in the newspaper article.
20	b	The report that had been written by the professors surprisingly was not mentioned in the newspaper article.
20	c	The report that had been written by the professor surprisingly were not mentioned in the newspaper article.
20	d	The report that had been written by the professors surprisingly were not mentioned in the newspaper article.
21	a	The memo that had been written by the manager unfortunately was not read until it was too late.
21	b	The memo that had been written by the managers unfortunately was not read until it was too late.
21	c	The memo that had been written by the manager unfortunately were not read until it was too late.

21	d	The memo that had been written by the managers unfortunately were not read until it was too late.
22	a	The computer that had been assembled by the technician eventually was going to stop working.
22	b	The computer that had been assembled by the technicians eventually was going to stop working.
22	c	The computer that had been assembled by the technician eventually were going to stop working.
22	d	The computer that had been assembled by the technicians eventually were going to stop working.
23	a	The book that had been written by the scientist finally was accepted for publication by a major publishing firm.
23	b	The book that had been written by the scientists finally was accepted for publication by a major publishing firm.
23	c	The book that had been written by the scientist finally were accepted for publication by a major publishing firm.
23	d	The book that had been written by the scientists finally were accepted for publication by a major publishing firm.
24	a	The movie that had been reviewed by the critic finally was approved for general viewing.
24	b	The movie that had been reviewed by the critics finally was approved for general viewing.
24	c	The movie that had been reviewed by the critic finally were approved for general viewing.
24	d	The movie that had been reviewed by the critics finally were approved for general viewing.

2. Log-transformed analyses

Note: results that differ from those reported in the main paper in terms of statistical significance are presented in gray.

2.1 Experiment 1 analysis with log-transformed reading times

	Regions											
	Pre-critical			Critical			Spillover 1			Spillover 2		
	$\hat{\beta}$	SE	t	$\hat{\beta}$	SE	t	$\hat{\beta}$	SE	t	$\hat{\beta}$	SE	t
**PP attractor**												
Grammaticality	-0.01	0.01	-1.47	-0.00	0.00	-0.07	**0.02**	**0.00**	**3.15**	0.01	0.00	1.56
Attractor number	0.01	0.01	1.36	0.00	0.00	0.47	**0.02**	**0.00**	**2.88**	0.01	0.00	1.32
Grammaticality × attractor number	-0.00	0.01	-0.47	-0.00	0.00	-0.13	0.06	0.00	0.81	**0.02**	**0.00**	**2.60**
Attraction	0.01	0.01	0.84	0.00	0.01	0.22	**0.02**	**0.01**	**2.53**	**0.03**	**0.01**	**2.80**
**Direct object attractor**												
Grammaticality	-0.01	0.01	-1.68	0.01	0.00	1.49	**0.04**	**0.00**	**4.71**	0.01	0.00	1.74
Attractor number	-0.00	0.01	-0.28	-0.00	0.00	-0.53	0.00	0.00	0.93	0.01	0.00	1.58
Grammaticality × attractor number	0.01	0.01	1.34	0.00	0.00	0.22	0.00	0.00	0.02	0.00	0.00	0.15
Attraction	0.01	0.01	0.78	-0.00	0.01	-0.19	0.00	0.01	0.61	0.01	0.01	1.15
Attraction × grammatical function	0.00	0.01	0.03	0.00	0.01	0.03	0.01	0.01	1.14	0.02	0.01	1.35

2.2 Experiment 2 analysis with log-transformed reading times

	Regions											
	Pre-critical			Critical			Spillover 1			Spillover 2		
	$\hat{\beta}$	SE	*t*	$\hat{\beta}$	SE	*t*	$\hat{\beta}$	SE	*t*	$\hat{\beta}$	SE	*t*
**PP attractor**												
Grammaticality	0.00	0.00	0.53	**0.02**	**0.00**	**2.36**	**0.05**	**0.00**	**5.65**	**0.01**	**0.00**	**2.39**
Attractor number	0.00	0.00	1.01	0.00	0.00	0.25	0.00	0.00	0/04	-0.00	0.00	-0.71
Grammaticality × attractor number	-0.00	0.00	-0.53	0.00	0.00	0.52	0.00	0.00	0.18	-0.00	0.00	-0.34
Attraction	0.00	0.01	0.34	0.00	0.01	0.56	0.00	0.01	0.17	-0.00	-0.01	-0.70
**Direct object attractor**												
Grammaticality	-0.00	0.00	-0.93	**0.00**	**0.00**	**4.29**	**0.05**	**0.00**	**7.39**	**0.04**	**0.00**	**6.77**
Attractor number	-0.00	0.01	-0.92	0.00	0.00	0.00	0.00	0.00	0.86	0.00	0.00	1.42
Grammaticality × attractor number	-0.01	0.00	-1.23	0.00	0.00	-0.30	-0.00	0.00	0.71	**-0.01**	**0.00**	**-2.77**
Attraction	-0.02	0.01	-1.69	0.00	0.01	-0.27	0.00	0.01	0.08	-0.00	0.00	-0.89
Attraction × grammatical function	0.02	0.01	1.46	0.01	0.01	0.63	0.00	0.01	0.06	0.00	0.01	0.07

2.3 Experiment 3 analysis with log-transformed reading times

	Regions											
	Pre-critical			Critical			Spillover 1			Spillover 2		
	$\hat{\beta}$	SE	*t*	$\hat{\beta}$	SE	*t*	$\hat{\beta}$	SE	*t*	$\hat{\beta}$	SE	*t*
**PP attractor**												
Grammaticality	0.01	0.00	1.71	0.00	0.00	-0.29	**0.05**	**0.00**	**7.98**	0.02	**0.00**	3.51
Attractor number	-001	0.00	-1.26	-0.00	0.00	-0.31	0.00	0.00	0.22	0.00	0.00	0.30
Grammaticality × attractor number	0.00	0.00	0.57	-0.00	0.00	-0.59	-0.00	0.00	0.60	-0.00	0.00	-0.29
Attraction	-0.01	0.01	-1.09	0.00	0.01	0.20	0.00	0.01	0.47	0.00	0.00	0.45
